# Evidence of efficient stop codon readthrough in four mammalian genes

**DOI:** 10.1093/nar/gku608

**Published:** 2014-07-10

**Authors:** Gary Loughran, Ming-Yuan Chou, Ivaylo P. Ivanov, Irwin Jungreis, Manolis Kellis, Anmol M. Kiran, Pavel V. Baranov, John F. Atkins

**Affiliations:** 1 School of Biochemistry and Cell Biology, University College Cork, Cork, Ireland; 2 CSAIL, Massachusetts Institute of Technology, Cambridge, MA 02139-4307, USA; 3 Department of Human Genetics, University of Utah, Salt Lake City, UT 84112-5330, USA

## Abstract

Stop codon readthrough is used extensively by viruses to expand their gene expression. Until recent discoveries in *Drosophila*, only a very limited number of readthrough cases in chromosomal genes had been reported. Analysis of conserved protein coding signatures that extend beyond annotated stop codons identified potential stop codon readthrough of four mammalian genes. Here we use a modified targeted bioinformatic approach to identify a further three mammalian readthrough candidates. All seven genes were tested experimentally using reporter constructs transfected into HEK-293T cells. Four displayed efficient stop codon readthrough, and these have UGA immediately followed by CUAG. Comparative genomic analysis revealed that in the four readthrough candidates containing UGA-CUAG, this motif is conserved not only in mammals but throughout vertebrates with the first six of the seven nucleotides being universally conserved. The importance of the CUAG motif was confirmed using a systematic mutagenesis approach. One gene, *OPRL1*, encoding an opiate receptor, displayed extremely efficient levels of readthrough (∼31%) in HEK-293T cells. Signals both 5′ and 3′ of the *OPRL1* stop codon contribute to this high level of readthrough. The sequence UGA-CUA alone can support 1.5% readthrough, underlying its importance.

## INTRODUCTION

In some organisms, in an mRNA-independent manner, UAG, UAA or UGA are reassigned from stop to specify an amino acid (1). In contrast, dynamic mRNA-specific redefinition of stop codons (2) is often influenced by mRNA context features that increase the chances of near-cognate tRNA successfully competing with release factor for ribosomal A-site acceptance. In the form of stop codon redefinition where the non-universal amino acids selenocysteine or pyrrolysine are specified, the identity of the amino acid encoded is of key functional significance. In other cases, commonly known as stop codon readthrough, the identity of the amino acid specified is unimportant and the key feature is the downstream encoded C-terminal extension. Until recently the repertoire of documented stop codon readthrough cases was limited mostly to viral genes where several cases have been described (3–7). Comparative sequence analysis and ribosome profiling studies are now, however, showing that readthrough is much more widespread in chromosomal gene expression than was previously appreciated.

Bioinformatic analysis of orthologous genes from 12 diverse *Drosophila* genomes revealed protein coding signatures in the zero frame downstream of the annotated stop codon for 283 protein coding genes (8,9). Some of these were experimentally tested and verified as bona fide readthrough cases (9) and three had been previously characterized. One experimentally studied case of developmentally regulated readthrough in *Drosophila* is in decoding of the *kelch* gene which is essential for the organization of the female ring canal actin cytoskeleton (10). Readthrough of the UGA stop codon in *kelch* extends the standard 76 kDa protein product to 160 kDa. The proportion of extended product is commonly ∼5% but in imaginal discs during metamorphosis, it is at a higher level (11).

The phylogenetic analyses of the 12 *Drosophila* genomes were by their nature designed to identify signatures of evolutionarily conserved readthrough. Therefore, they did not reveal instances of readthrough that either emerged recently or are not under strong evolutionary selection. This gap in our knowledge has recently been filled by ribosome profiling analysis. Deep sequencing of ribosome-protected mRNA fragments of *Drosophila* *melanogaster* early embryos, and S2 tissue culture cells, provided experimental evidence for over 300 readthrough events not predicted by phylogenetic approaches; and for some of these, functional domains in the extensions have already been demonstrated (12). A number of these instances of readthrough appear to be developmentally regulated; many more will doubtless be identified when other tissues are examined. The more modest number identified to date in [*PSI^−^*] *Saccharomyces cerevisiae* (12–14) likely does not reveal the full importance of readthrough in that organism since the balance of various factors, and most notably [*PSI^+^*], liberates evolutionarily important diversity (15–17). [The [*PSI^+^*] variant of termination factor eRF3 enhances readthrough (review, 18).]

Analysis of stop codon readthrough in mammalian gene expression is at an early stage. Phylogenetic analysis predicted four candidates, *SACM1L*, *OPRK1*, *OPRL1* and *BRI3BP* (9,19), and a readthrough isoform of myelin P0 was studied experimentally (20).

While the identity of the nucleotide 3′ adjacent to a stop codon alone has a substantial effect on termination stringency (21–23), the context effect is not limited to a single nucleotide. This is known to be exploited by some readthrough cases to elevate its efficiency. The 3′ hexanucleotide, CARYYA, is important for synthesizing the 57 kDa readthrough extension to a Tobacco Mosaic Virus protein (24). It also stimulates readthrough in many other cases, especially in plant viruses (25,26). A similar sequence (CARNBA) can stimulate readthrough in yeast (27).

At an early stage a 3′ stem loop was identified for a case of *Drosophila* readthrough (28). Interestingly in Barley Yellow Dwarf Virus, there is a stimulatory structure that involves base pairing between long distant segments, >700 nucleotides 3′ of the UAG stop codon (4). Stimulation of readthrough by 3′ mRNA structures is much more commonly utilized than was appreciated until recently, for example in *Drosophila* kelch (6). Further, 3′ adjacent stem loops are involved in a subset of cases of eukaryotic selenocysteine specification (29,30). RNA pseudoknot structures are also able to stimulate readthrough as in the case of Murine Leukemia Virus (MuLV) GagPol (31). The MuLV pseudoknot stimulation of readthrough involves a change in its conformation (32).

There is a highly conserved stem loop structure of high potential stability 3′ of the stop codon in the human *SACM1L* (7). However, studies in a heterologous expression system suggested very low levels of readthrough at *SACM1L* mRNA (33).

Sequences 5′ of stop codons are also relevant, in part because of effects at the nascent peptide level. A major effect of C-terminal proline on termination efficiency has been revealed by many studies (e.g. 34). Earlier systematic studies are also relevant for their insights into the effects that the identity of the last two amino acids of the nascent polypeptide have on termination efficiency in bacteria (35). However, as there are substantial differences in context effects in bacteria and eukaryotes (21), the 5′ context effect studies in both *S. cerevisiae* (36) and mammalian cells (37) are more relevant to the discovery of the new natural stimulatory motif reported here.

The present work experimentally tests readthrough of the published human candidates, *SACM1L*, *OPRK1*, *OPRL1* and *BRI3BP* (9) as well as three new candidates identified here.

## MATERIALS AND METHODS

### Plasmids

Sequences flanking the stop codons of the seven predicted readthrough candidates were chemically synthesized (Integrated DNA Technologies: g blocks) and cloned with XhoI and BglII restriction enzymes into pDluc dual-luciferase vector. The *SACM1L* (NM_014016.3) insertion sequence range was 1950–2061 with a T to C mutation at 2032 to remove the second stop codon while still maintaining the predicted secondary structure. The sequence boundaries of the remaining six candidates were as follows: *ACP2* (NM_001610.2) 1372–1419; *MAPK10* (NM_138982.2) 2064–2120; *BRI3BP* (NM_080626.5) 876–989; *AQP4* (NM_001650.4) 1017–1119; *OPRK1* (NM_000912.3) 1348–1462; *OPRL1* (NM_182647.2) 1511–1616. Mutation constructs were derived by two-step polymerase chain reaction with appropriately designed primers (Integrated DNA Technologies). Corresponding readthrough controls were generated for every reporter construct by mutating TGA stop codons to TGG or else TAA and TAG stop codons to TAC (Supplementary Figure S5C).

HA-AQP4-TGA and HA-AQP4-TGG were made by Gibson Assembly (NEB) using three overlapping synthetic DNA fragments (Integrated DNA Technologies: g blocks) based on the AQP4 CDS (NM_001650.4). Assembled gene fragments were cloned in-frame with the influenza hemagglutinin tag in pcDNA3-HA (Invitrogen). All constructs were verified by DNA sequencing.

### Cell culture and transfections

HEK-293T cells (ATCC) were maintained in Dulbecco's modified Eagle's medium (DMEM) supplemented with 10% FBS, 1 mM L-glutamine and antibiotics. HEK-293T cells were transfected in quadruplicate with Lipofectamine 2000 reagent (Invitrogen), using the 1-day protocol in which suspended cells are added directly to the DNA complexes in half-area 96-well plates. For each transfection the following were added to each well: 25 ng of each plasmid plus 0.2 μl Lipofectamine 2000 in 25 μl Opti-Mem (Gibco). The transfecting DNA complexes in each well were incubated with 4 × 10^4^ cells suspended in 50 μl DMEM + 10% FBS. Transfected cells were incubated at 37°C in 5% CO_2_ for 24 h.

### Dual-luciferase assay

Firefly and Renilla luciferase activities were determined using the Dual Luciferase Stop & Glo® Reporter Assay System (Promega). Relative light units were measured on a Veritas Microplate Luminometer with two injectors (Turner Biosystems). Transfected cells were washed once with 1 x PBS and then lysed in 12.6 μl of 1x passive lysis buffer (PLB) and light emission was measured following injection of 25 μl of either Renilla or firefly luciferase substrate. Readthrough efficiencies (% readthrough) were calculated by averaging relative luciferase activities (firefly/Renilla) from replicate wells of test constructs and dividing by average luciferase activities from replicate wells of control constructs (stop mutated to sense). Readthrough efficiencies of at least three independent experiments are graphed.

### Western analysis

Cells were transfected in 6-well plates using Lipofectamine 2000 reagent, again using the 1-day protocol described above, with 3 μg of each indicated plasmid. The transfecting DNA complexes in each well were incubated with 2.4 × 10^6^ HEK-293T cells suspended in 3000 μl DMEM + 10% FBS and incubated overnight (3 days for HA-AQP4 transfections) at 37°C in 5% CO_2_. Transfected cells were lysed in 100 μl 1x PLB. For HA-AQP4 transfections cells were lysed in subcellular fractionation buffer (250 mM sucrose, 20 mM HEPES (pH 7.4), 10 mM KCl, 1.5 mM MgCl_2_, 1 mM EDTA, 1 mM EGTA and 1 mM DTT). Proteins were resolved by sodium dodecyl sulphate-polyacrylamide gel electrophoresis and transferred to nitrocellulose membranes (Protran), which were incubated at 4°C overnight with primary antibodies. Immunoreactive bands were detected on membranes after incubation with appropriate fluorescently labeled secondary antibody using a LI-COR Odyssey® Infrared Imaging Scanner.

### Antibodies

An affinity purified rabbit polyclonal antibody to a predicted antigen (DRTESRQDSLELSS) within ORF2 of AQP4 was prepared by GenScript. The following commercially available antibodies were also used. Mouse anti-Renilla (MBL), mouse anti-HA (Covance), mouse anti-β-actin (Sigma) and goat anti-AQP4 (Santa Cruz).

## RESULTS

### Phylogenetic analysis reveals evolutionary constraint 3′ of the stop codon in seven human genes

Previous searches for evolutionary conserved readthrough cases in humans identified four potential candidates: *SACM1L*, *OPRL1*, *OPRK1* and *BRI3BP* (9,19). Because all four of these candidates have highly conserved UGA stop codons, as do the vast majority of readthrough candidates predicted in other metazoa (9), we searched for additional candidates specifically among genes with highly conserved UGA stop codons using PhyloCSF (38) with a correspondingly relaxed multiple-hypothesis correction. PhyloCSF uses substitutions and codon frequencies among 29 mammals to distinguish coding from non-coding regions irrespective of nucleotide-level conservation. We found three additional candidates using this method, *ACP2*, *AQP4* and *MAPK10* (Supplementary Figure S1A–C). Each of the seven candidate readthrough extensions has a PhyloCSF score higher than 99.96% of similar-sized non-coding regions of 3′-UTRs (Supplementary Figure S2). We used RNAz (39) to predict that *ACP2* has a 42-nucleotide conserved RNA stem loop starting six nucleotides 3′ of the predicted readthrough stop codon (Supplementary Figure S1D). RNAz did not predict conserved RNA secondary structures in either *AQP4* or *MAPK10*. To help define sequences that could potentially stimulate readthrough we sought to identify non-mammalian orthologs of the seven readthrough candidates. For *AQP4, MAPK10, OPRK1*, *OPRL1* and *SACM1L*, conserved C-terminal extensions were observed beyond mammals (Supplementary Figure S3A–E). In *AQP4* and *SACM1L*, the conservation extends to at least the last common ancestor of mammals and cartilaginous fishes. For *MAPK10* the extension is conserved to at least the last common ancestor of mammals and lampreys and in *OPRK1* and *OPRL1* it goes to at least the last common ancestor of mammals and bony fishes.

**Figure 1.**
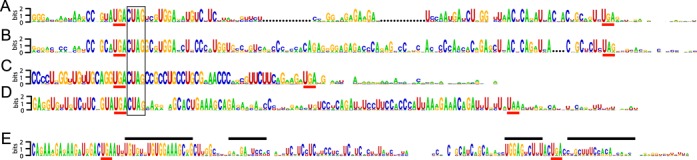
WebLogo representation showing the nucleotide conservation of the five readthrough candidates examined. Representation: (**A**) for *OPRK1*, (**B**) for *OPRL1*, (**C**) for *MAPK10*, (**D**) for *AQP4* and (**E**) for *SACM1L*. Dotted areas in A and B represent gaps in the alignment between *OPRK1* and *OPRL1*. The stop codons of the main ORFs and the readthrough extensions are underlined in red. The regions in *SACM1L* mRNA proposed to be involved in a stem-loop secondary structure are indicated by black bars above the conservation plot in E. The highly conserved tetranucleotide motif CUAG following the main ORF stop codon in *OPRK1*, *OPRL1*, *AQP4* and *MAPK10* is boxed.

*OPRL1* and *OPRK1* are paralogs encoding G protein-coupled opioid receptors. The amino acid sequences of their respective extensions, except for six of the last eight residues, show little similarity between the paralogs (See Weblogo (40) in Figure 1A and B, Supplementary Figure S4). However, the extensions in *OPRK1* homologs are almost always exactly 28 residues long and conservation at the amino acid level is strongest near the C-terminus (Supplementary Figures S3C and S4). The extensions in *OPRL1* also show high amino acid conservation near the C-terminus but their lengths vary from 28 to 34 residues with the deletions and insertions concentrated in the central region (Supplementary Figure S3D). The nucleotide sequences surrounding the stop codon of the main open reading frames (ORFs) in *OPRK1* and *OPRL1* exhibit a striking level of conservation (Supplementary Figure S3C and D). The sequence 5′ of the stop codon shows conservation primarily at the amino acid level although the last sense codon is only moderately conserved—predominately GUA in *OPRK1* and GCA in *OPRL1*. The stop codon is always UGA. The four nucleotides immediately 3′ of the UGA stop codon—CUAG—are universally conserved within the two paralogous groups and their respective orthologs. In *OPRL1*, but not *OPRK1*, the next two nucleotides, GC, are also absolutely conserved and the following 12 nucleotides are moderately conserved, the first six of them clearly primarily at the nucleotide level. No other sequences 3′ appear conserved strictly at the nucleotide level alone in either *OPRK1* or *OPRL1*. The features of the *OPRL1* and *OPRK1* extension described above suggest that their readthrough capability predates their divergence.

MAPK10 is a member of the mitogen activated protein kinase family. Its readthrough extension is always 13 residues long (Supplementary Figure S1C). The penultimate and the last sense codons of the first ORF encoding this protein are always the same—UGC-AGG. The three nucleotides following the stop codon are always CUA and in more than 90% (68 of 73 cases) the next nucleotide is G, forming the same tetranucleotide sequence, CUAG that is universally conserved at the same position in *OPRK1* and *OPRL1* (Figure 1 and Supplementary Figures S1 and S3). The next eight nucleotides are also highly conserved, primarily at the nucleotide level and not the amino acid level. Seven of the nucleotides in this region have the potential to base pair with conserved nucleotides of the last two sense codons and the stop codon of ORF1.

AQP4 belongs to the aquaporin family of membrane proteins and is involved in conducting water through the cell membrane in kidneys and cells of neuronal origin. The readthrough extension of AQP4 is typically 28 residues (Supplementary Figure S1B). Like OPRK1 and OPRL1, conservation of the extension at the amino acid level is concentrated near the C-terminus. Unlike in *OPRK1* and *OPRL1* the last sense codon of the first ORF is always the same—GUA (Figure 1D and Supplementary Figure S3A). Remarkably, the four nucleotides following the stop codon, CUAG, are highly conserved, with the first three universally conserved, and are identical to the universally conserved nucleotides in the same position in *OPRK1*, *OPRL1* and *MAPK10* genes. Of the other nucleotides 3′ of notable conservation are those present at positions 13–24 after the stop codon. However, only two of them are absolutely conserved.

Suppressor of actin 1 (SACM1L) is a membrane associated protein. Its readthrough extension appears highly variable in size, from 19 to 38 residues (Supplementary Figure S3E). The four C-terminal amino acids are highly conserved. Like *AQP4* and *MAPK10* the last sense codon of the first ORF is always the same—GAC. We previously proposed that the sequence 3′ of the stop codon of *SACM1L*, at least in mammals, can be folded in an energetically stable RNA secondary structure that potentially can be involved in stimulating the readthrough (9,19). The present analysis including sequences from non-mammalian vertebrates indicates that the proposed RNA secondary structure, as previously proposed, is only conserved in mammals. A homologous putative structure, supported by co-variant nucleotide substitutions, lacking part of one stem, is present throughout vertebrates, including cartilaginous fishes and coelacanth, but is completely absent from ray-finned fishes.

### Experimental evidence of readthrough for *OPRK1*, *OPRL1*, *MAPK10* and *AQP4*

**Figure 2.**
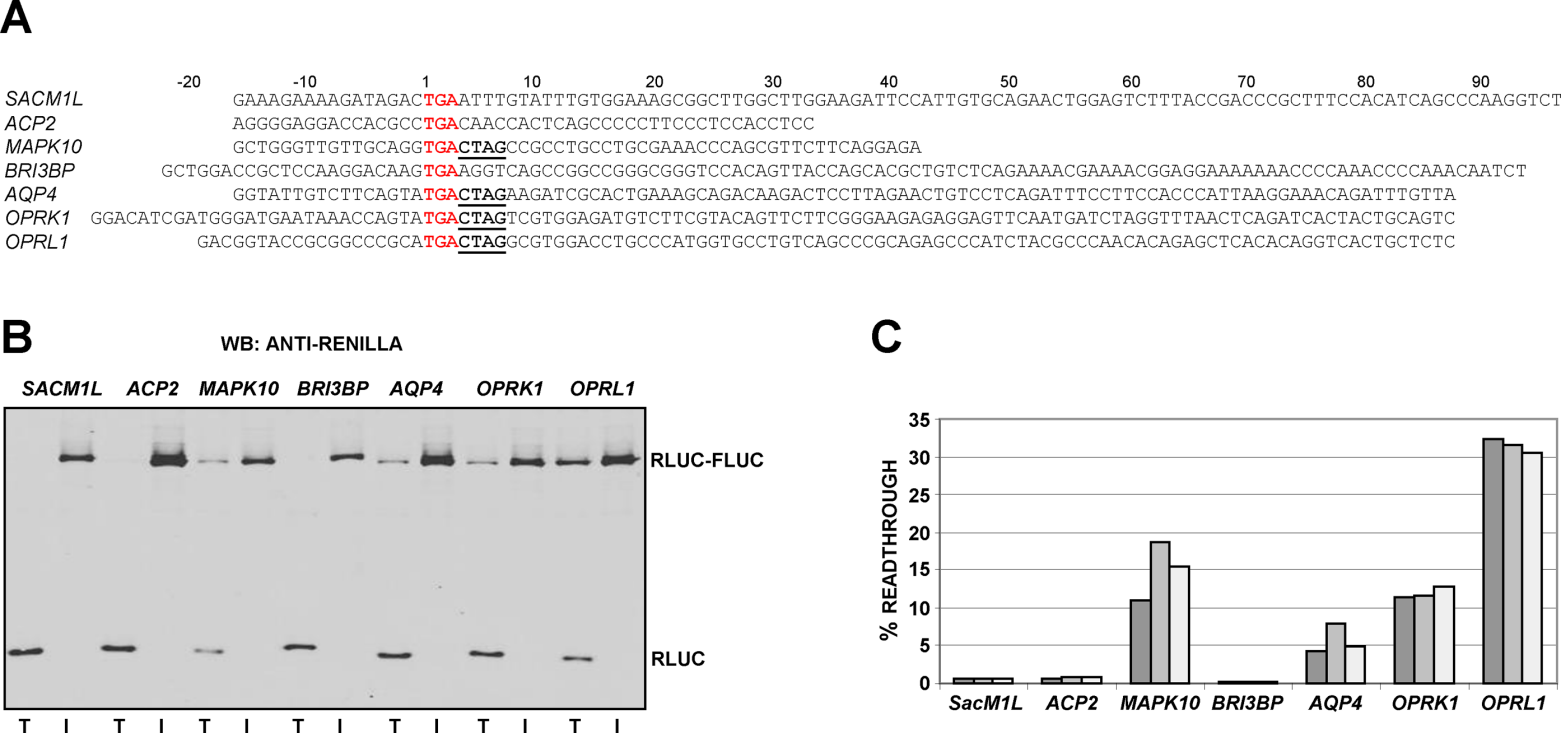
(**A**) Sequence boundaries of seven readthrough candidates cloned into a dual-luciferase plasmid. Main ORF stop codons are in red font and the conserved CTAG motif is highlighted in bold and underlined. Plasmids were transfected into HEK-293T cells and 24 hr later lysates analysed for readthrough by both western blotting with anti-Renilla (**B**) and dual-luciferase assay to determine readthrough efficiencies (**C**). For (B) each TGG in-frame control (I) is adjacent to its respective test (T) construct. For (C) readthrough efficiencies were calculated as the ratio of test construct luciferase activity to its TGG in-frame control from three independent experiments.

To verify the functionality of the seven predicted readthrough candidates, local sequences for each (see Figure 2A for boundaries) were cloned in-frame between Renilla and firefly luciferase genes. Lysates from HEK-293T cells transfected with each construct were assayed for luciferase activities and expression levels. The 3′ firefly luciferase gene lacks an initiation codon and its expression is dependent on readthrough of the candidate's stop codon. Readthrough efficiencies were determined by comparing relative luciferase activity (firefly/Renilla) of test constructs with controls for each construct in which the UGA stop codon is changed to UGG (TRP). Efficient stop codon readthrough was observed for the candidates *OPRL1*, *OPRK1*, *MAPK10* and *AQP4* by western blotting with anti-Renilla luciferase antibodies (Figure 2B). Readthrough efficiencies were determined by dual-luciferase assay and were as follows: ∼31% (*OPRL1*), ∼13% (*OPRK1*), ∼14% (*MAPK10*) and ∼7% (AQP4) (Figure 2C). *SACM1L*, *ACP2* and *BRI3BP* stop codon cassettes failed to promote levels of readthrough greater than 1% in these cells.

### Identification of a conserved 3′ readthrough motif

**Figure 3.**
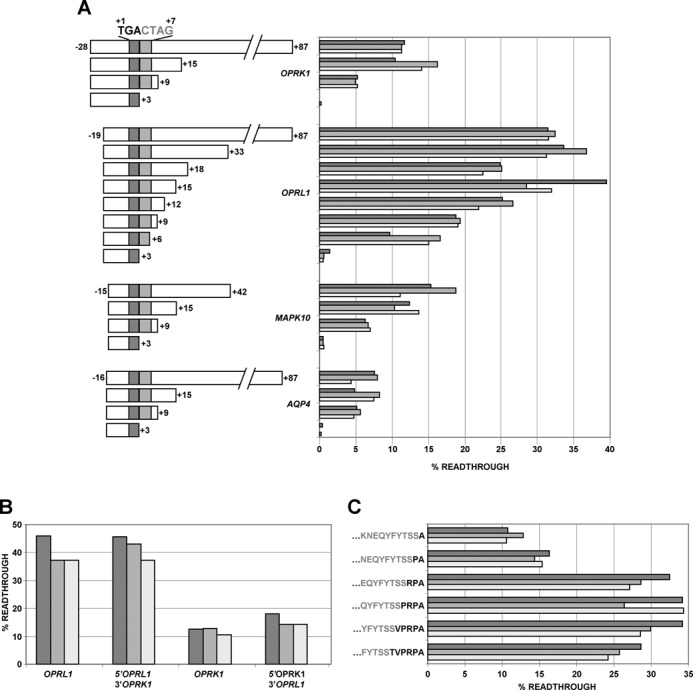
(**A**) Readthrough efficiency of dual luciferase constructs with 3′ nested deletions of *OPRK1, OPRL1*, *MAPK10* and *AQP4*. (**B**) 5′ and 3′ sequences of *OPRL1* and *OPRK1* swapped and readthrough efficiencies determined. (**C**) Analysis of the effect of sequences encoding the nascent peptide on the readthrough efficiency of *OPRL1*. Amino acids in gray-type face are encoded within the plasmid (C-terminus of Renilla) and those in bold type face are from OPRL1.

Though context dependent, several studies have shown UGA to be the leakiest of the three stop codons. The annotated stop codon of all seven readthrough candidates is UGA, however, strikingly, only those candidates confirmed here experimentally (*OPRK1*, *OPRL1*, *MAPK10* and *AQP4*) contain the highly conserved CUAG tetranucleotide sequence immediately 3′ of their annotated UGA stop codons as described above (boxed in Figure 1 and underlined in Figure 2A). To assess the possible role of nucleotides 3′ of the stop codon on readthrough we made several sets of 3′ nested deletions for *OPRK1*, *OPRL1*, *MAPK10* and *AQP4* readthrough. For all four candidates, when the CUAG motif was completely deleted, readthrough levels became almost undetectable (Figure 3A). While there are several instances of readthrough stimulation by RNA secondary structures 3′ of the stop codon, here, only 12 nucleotides 3′ of the stop codon (+15 in Figure 3A) appears to be sufficient for maximal readthrough of *OPRK1*, *OPRL1*, *MAPK10* and *AQP4*. Though deletion of the CUAG motif results in the most dramatic decrease in readthrough efficiency for each of the readthrough genes, deletion of nucleotides +9 to +15 in *OPRK1*, *OPRL1* and *MAPK10* results in ∼2-fold decrease in readthrough efficiency.

### Analysis of *OPRL1* 5′ sequences

Because of the relatively high level of readthrough observed for *OPRL1* (∼31%), we decided to focus on this candidate for systematic mutational analysis to delineate local sequence requirements effecting readthrough levels. The only difference between the 12 nucleotides immediately 3′ of both *OPRK1* and *OPRL1* annotated coding regions are two nucleotides at positions +8 and +15 (where the first nucleotide of the UGA stop codon is assigned +1), yet the readthrough efficiency for *OPRL1* is almost 2.5 times greater than that for *OPRK1* (∼31% versus ∼13%). Swapping the 12 nucleotides immediately 3′ of *OPRL1* with those of *OPRK1* did not reduce readthrough levels of *OPRL1* to those of *OPRK1* levels (Figure 3B). Similarly, swapping the 12 nucleotides 3′ of *OPRK1* with those of *OPRL1* did not increase the efficiency of *OPRK1* readthrough. This suggests that *OPRL1* sequences 5′ of the main ORF stop codon are responsible for the difference in readthrough levels between *OPRL1* and *OPRK1*.

Complete deletion of the *OPRL1* 5′ sequences reduced readthrough levels to ∼5% when 87 3′ nucleotides are present (Supplementary Figure S5A). Together these results indicate that while the CUAG motif is important for readthrough, an additional stimulation is exerted by sequences 5′ of the UGA stop codon. In the case of *OPRL1*, this additional stimulation is ∼5-fold (since readthrough decreases from ∼31% to ∼5% when *OPRL1* 5′ sequences are deleted).

The sequential restoration of *OPRL1* codons 5′ of UGA had dramatic effects on readthrough efficiency with only six codons required to regain maximum *OPRL1* readthrough (Figure 3C). The addition of just one codon 5′ of the *OPRL1* stop codon resulted in ∼50% restoration in readthrough efficiency (cf >10% readthrough in Figure 3C to ∼5% readthrough in Supplementary Figure S5A when the last codon is not GCA). The nucleotide immediately 5′ of readthrough stop codons has been shown previously by others (41) to have an influence on readthrough efficiency and in agreement with this we see that a purine in this position is preferable (Supplementary Figure S5B).

### Analysis of *OPRL1* 3′ sequences

**Figure 4.**
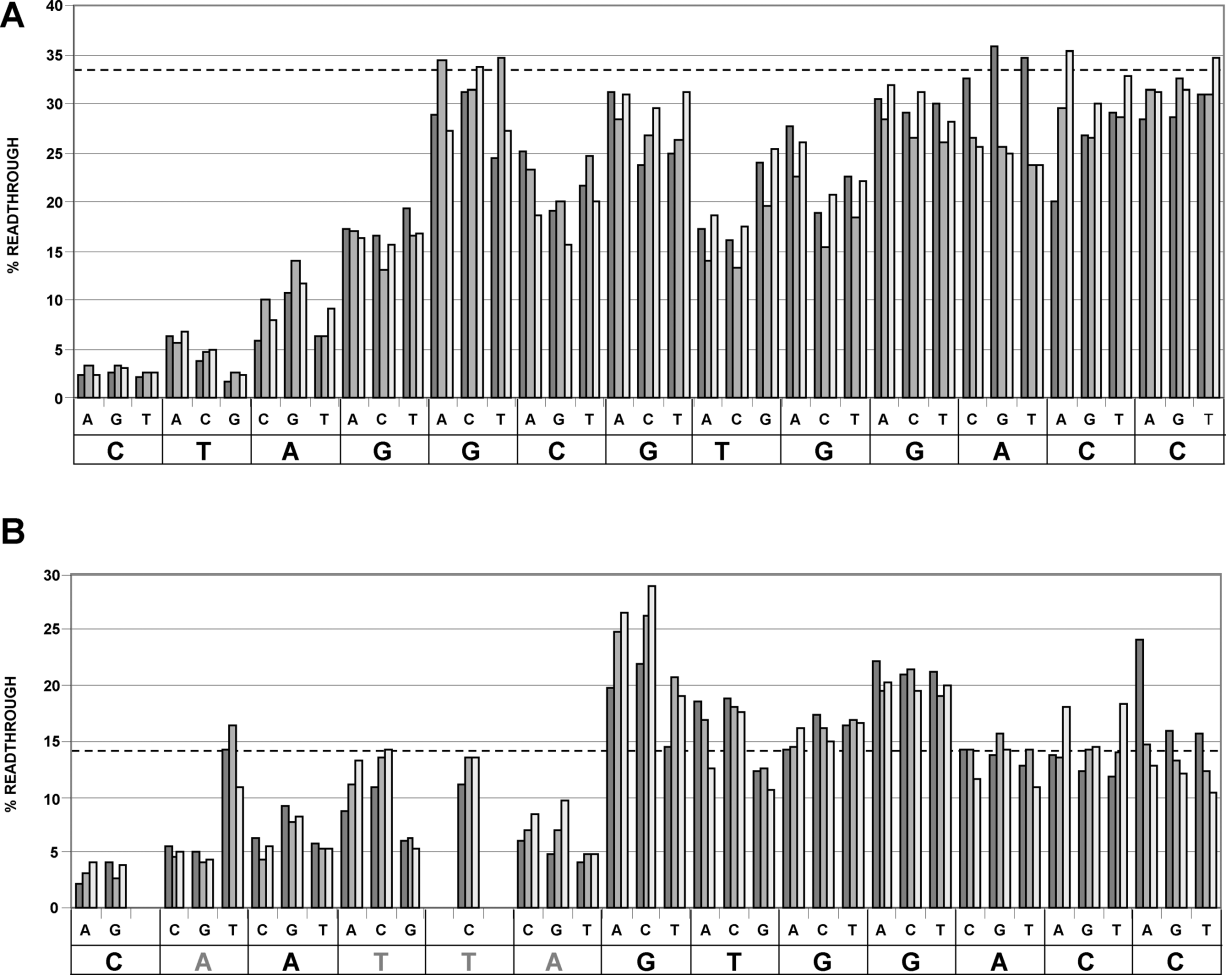
Systematic mutational analysis of the 13 nucleotides immediately 3′ of *OPRL1* TGA (**A**) and *OPRL1*-CARYYA TGA (**B**). The dotted lines represent the mean readthrough efficiencies of the wild-type *OPRL1* (A) and *OPRL1*-CARYYA (B). Sequences at the bottom of each graph are those of the 13 nucleotides immediately 3′ of *OPRL1* TGA (A) and *OPRL1*-CARYYA TGA (B). The triplet of nucleotides immediately above each nucleotide of these sequences represent the substitution mutations (those in B introducing a zero frame stop codon were excluded).

To better understand the importance of each nucleotide 3′ of the UGA stop codon, we systematically mutated each of the thirteen 3′ nucleotides to each of the other three possible nucleotides in turn and then assessed readthrough efficiency by dual-luciferase assay (Figure 4A). Substitutions furthest 3′ of the UGA stop codon had the least effect on readthrough whereas those immediately 3′-adjacent had the most dramatic outcome. As reported previously for UGA readthrough (22,23), a cytosine immediately 3′ is especially important, however, in the *OPRL1* context the identity of the following three nucleotides 3′ of the cytosine are also critical. Most other mutations in this series had only modest effects on *OPRL1* readthrough.

Another readthrough motif, CARYYA, also immediately 3′ of the stop codon, has been reported previously to stimulate readthrough (24,26). We investigated whether the CARYYA motif could replace the hexanucleotide sequence of *OPRL1* when placed immediately 3′ of the stop codon. The readthrough efficiency of *OPRL1*-CARYYA is ∼15% (Figure 4B) indicating that, at least in the *OPRL1* context, CARYYA is a less efficient readthrough stimulator than the CUAG motif. Systematic mutational analysis of the *OPRL1*-CARYYA confirmed that all six nucleotides of the CARYYA appear to be important for readthrough (Figure 4B).

### Stop codon identity is important for the CUA_G readthrough motif

To ascertain the importance, if any, of stop codon identity 5′ of the CUAG and CARYYA motifs we generated readthrough constructs with wild-type *OPRL1* and *OPRLI*-CARYYA with either of the three stop codons. Changing the *OPRL1* stop codon from UGA to either UAA or UAG reduced readthrough efficiency by ∼6-fold and 3-fold, respectively, indicating that UGA is the most efficient readthrough stop codon with this new motif (Supplementary Figure S5C). In contrast, we find that for the CARYYA motif, readthrough efficiency is very similar with either UGA or UAG, both of which are much more efficient than UAA.

### Aminoglycoside effect on readthrough

**Figure 5.**
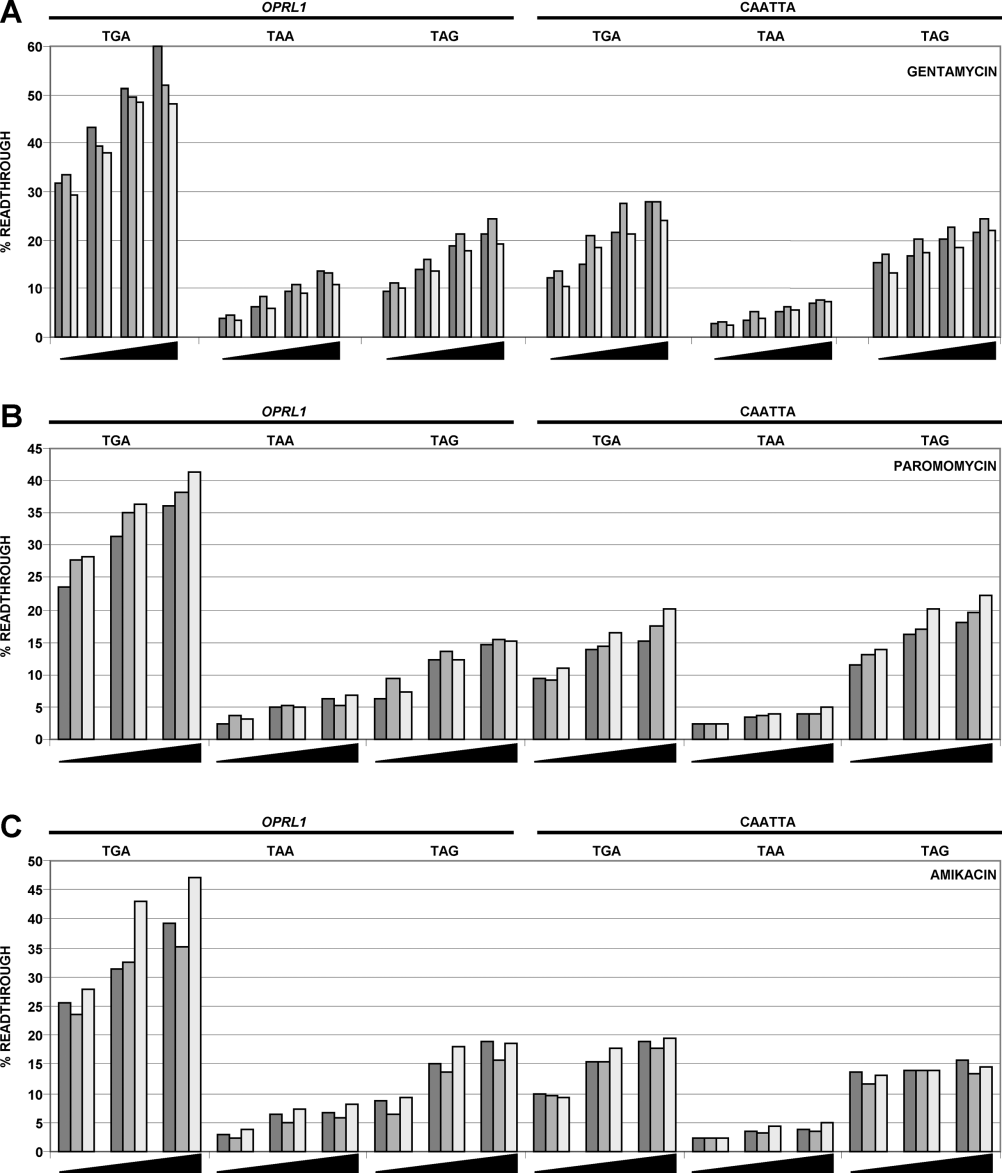
The effect of aminoglycoside addition to cells expressing the six constructs described in Supplementary Figure S5C. Aminoglycoside treatments included gentamycin (**A**), paromomycin (**B**) and amikacin (**C**).

Aminoglycoside antibiotics such as gentamycin are known to enhance readthrough of stop codons (42–47) and have been suggested as possible therapeutic interventions to suppress nonsense mutations (e.g. subsets of Duchene's Muscular Dystrophy and Cystic Fibrosis patients) (48,49). Early studies on aminoglycoside-induced readthrough indicate that readthrough efficiency may be influenced by the sequence context surrounding the stop codon (50,51) which could have implications for the treatment of patients with nonsense mutations in their DNA. We tested the effect of the aminoglycosides gentamycin, paromomycin and amikacin on readthrough of both wild-type *OPRL1* and *OPRL1*-CARYYA constructs with each of the three stop codons (Figure 5). For wild-type *OPRL1* constructs with either of the three stop codons, all aminoglycosides enhanced readthrough in a dose responsive manner. UGA readthrough was the most and UAA readthrough was the least responsive to aminoglycoside treatments. For *OPRL1*-CARYYA both gentamycin and amikacin had little effect on readthrough at UAG or UAA but did induce a dose-dependent increase at UGA stop codons. Paromomycin enhanced *OPRL1*-CARYYA readthrough on both UGA and UAG while having little effect on UAA stop codons. Together these results indicate that varying stop codons and 3′ contexts can respond dramatically to different aminoglycosides.

### Immunoblotting demonstrates readthrough in natural context

**Figure 6.**
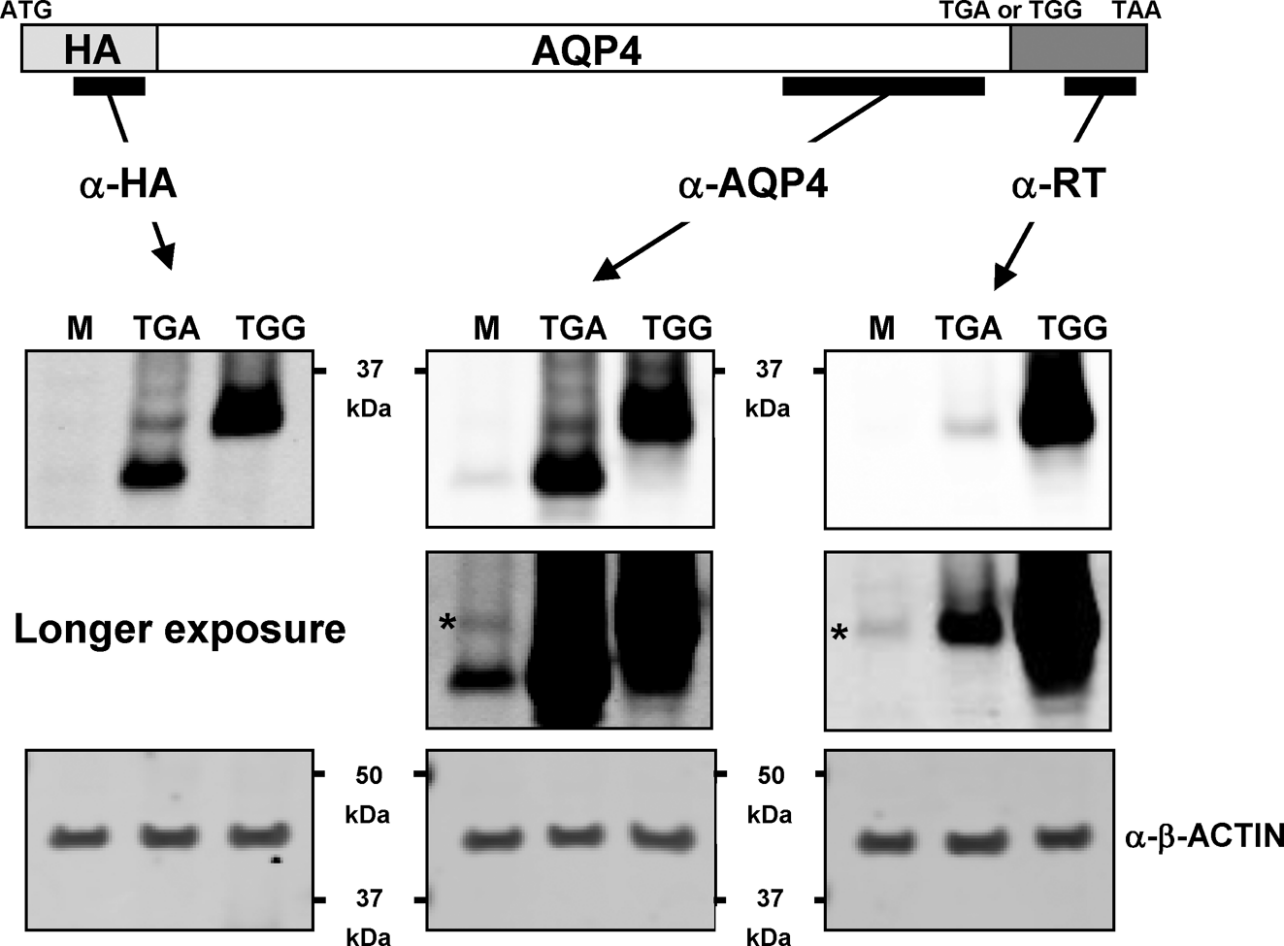
Immunodetection of AQP4 proteins. Western blots of protein lysates from HEK-293T cells either mock-transfected (M) or transfected with HA-AQP4-TGA (TGA) or HA-AQP4-TGG (TGG). α-RT is a custom made antibody raised against an AQP4 readthrough encoded epitope. * indicates immunodetection of putative endogenous AQP4 readthrough protein by α-RT.

In an attempt to identify readthrough for any of the four candidates in the context of the full coding sequence, we cloned each in-frame with sequences encoding an epitope tag (HA) so that expressed proteins have HA fused to their N-terminus. For each construct, sequences extending to the end of ORF2 were included. Constructs in which TGA was changed to TGG were also constructed so that the coding sequence and readthrough extension are expressed as a single ORF (readthrough control). Each construct was transfected into HEK-293T cells and lysates analysed by western blotting with antibodies against HA. HA-MAPK10 constructs were not well expressed and both HA-OPRL1 and HA-OPRK1 were expressed as multiple diffuse bands, making it difficult to identify potential readthrough products (not shown). However, HA-AQP4-TGA and HA-AQP4-TGG were well expressed and migrated as distinct proteins of ∼32 kDa (36.2 kDa predicted) and ∼35 kDa (39.6 kDa predicted), respectively (Figure 6, left-hand panel). For HA-AQP4-TGA a much fainter protein of ∼35 kDa is also evident. This protein co-migrates with the protein expressed from the HA-AQP4-TGG readthrough control. Furthermore, western analysis of the same lysates with a commercially available antibody against an AQP4 C-terminal epitope detected the same sized proteins as detected by the HA antibody (Figure 6, middle panels). Similar sized proteins are also evident in mock infected cells, suggesting that anti-AQP4 can detect endogenous expression of AQP4 and its readthrough product in these cells. However, AQP4 isoforms generated by leaky scanning are well documented (52) (leaky scanning does not explain the presence of putative readthrough proteins in cells expressing N-terminally HA-tagged AQP4-TGA since leaky scanning produces proteins with truncated N-termini).

To establish whether the putative AQP4 readthrough proteins are genuine, we raised antisera against an epitope within the predicted AQP4 readthrough extension. Immunoblotting of lysates transfected with constructs expressing either HA-AQP4-TGA or HA-AQP4-TGG detected only the ∼35 kDa protein, to varying levels, in both lysates (Figure 6, right-hand panels). Longer exposures revealed a protein of ∼35 kDa in mock infected cells that we assume is the endogenous AQP4 readthrough protein.

## DISCUSSION

Before the advent of ribosome profiling the majority of published stop codon readthrough examples were found in viral decoding (3–7). Until very recently only two examples of mammalian readthrough had been confirmed experimentally (20,53) although four more had been predicted by comparative genomic studies (9,19). A study published in 2013 using ribosome profiling identified 42 genes displaying stop codon readthrough in human foreskin fibroblasts (12), although the proportion that is productively utilized is unknown. None of the seven candidates studied here was expressed to sufficient levels to have been properly assessed by this ribosome profiling study. This may not be so surprising since *OPRL1*, *OPRK1* and *MAPK10* expression is known to be confined to neuronal cells whereas *AQP4* expression is predominantly in kidney and astrocyte cells. Here the prior comparative genomics analysis was extended leading to the identification of a further three mammalian readthrough candidates. We experimentally tested all seven and confirm readthrough in four.

TargetP analysis of the four readthrough examples did not reveal any cellular targeting motifs although AQP4, OPRL1 and OPRK1 all possess well-conserved serine and threonine residues (Supplementary Figure S4). AQP4, OPRL1 and OPRK1 are multiple pass transmembrane proteins where the readthrough extension would be predicted to reside in the cytoplasm. Serine and threonine residues could be sites of phosphorylation that may be involved in regulating desensitisation. Interestingly, readthrough of 3-phosphoglycerate in *Ustilago maydis*, a basidiomycete plant pathogen, reveals a functional peroxisomal targeting motif (54). The degree of importance of readthrough for generating dual targeted protein isoforms merits further investigation.

Factors affecting readthrough efficiency include tRNA abundance, RNA secondary structure, release factor levels, stop codon context and more recently posttranslational modifications of either the ribosome (55) or release factor (56). Since tRNA abundance and release factor levels are presumably invariant in our study and none of the confirmed readthrough genes have conserved RNA secondary structures it is most likely that context effects are the key determinants of readthrough efficiency for our candidates. Many groups have reported that readthrough is affected by the nucleotide sequence surrounding the stop codon (3,27,57–61). Mechanisms explaining the influence of downstream nucleotides and RNA secondary structures on the competition between productive near-cognate tRNA and release factor recognition of stop codons relevant to readthrough efficiency are still lacking. Several features have been considered including tRNA selection through stabilization of the A-site tRNA:mRNA interaction by stacking effects, interaction between the stop codon and the rRNA and interaction between the stop codon and the polypeptide chain release factor (62). Identity of the base 3′ adjacent to a stop codon was shown by binding studies to be important for release factor action (63). While the preferred termination signal differs in prokaryotic and eukaryotic species, a common feature appears to be a strong bias against a cytidine residue following a termination codon in all organisms (22,23), implying that any termination codon followed by a C is a weak stop codon. Dramatic recent advances in the resolution of ribosome structure using cryoEM will likely reveal information about the ribosome conformational changes that influence A-site selection in response to recoding signals.

*AQP4*, *MAPK10*, *OPRK1* and *OPRL1* all possess CUAG immediately 3′ of their termination codons and our mutational analyses of the *OPRLI* context clearly demonstrate the importance of these nucleotides. A search of all human genes for CUAG immediately following a UGA stop codon indicated that there were 23 instances (∼39 expected assuming equiprobable nucleotide occurrence). Four had positive PhyloCSF scores and these are the four candidates confirmed in this study. Efficient readthrough cannot be ruled out for those genes with UGA_CUAG and negative PhyloCSF scores since readthrough may be highly efficient but unimportant for organism fitness. However, one gene (Malate dehydrogenase; *MDH1*) had a borderline negative PhyloCSF score. We tested *MDH1* for readthrough using a dual-luciferase assay and confirmed ∼3.5% readthrough efficiency (not shown). This is the lowest level of readthrough that we observed for those candidates with CUAG 3′ of UGA stop codons and emphasises the importance of sequences extending beyond this motif. Interestingly, only one of the readthrough genes identified in the human foreskin fibroblast profiling study (12) possesses the UGA_CUAG stop codon context and this gene is *MDH1*.

Several groups have classified readthrough stop codons based on both nucleotide context and the presence of a 3′ RNA secondary structure (25,26). One type is generally made up of diverse plant viruses containing UAG_CARYYA or UAG_CARNBA in yeast (27). Another class generally has a pseudoknot structure 3′ of UGA_G. Another set made up mostly of animal viruses has UGA followed by either CGG or CUA. Only a handful of viruses (alphaviruses) have UGA_CUA and these include Sinbis, Middleburg (UGA_CUAG), Ross river and Getah (UGA_CUAGGC that is the same sequence as *OPRL1*) and also Chikungunya (UGA_CUAG) that is dangerously spreading. UGA_CUA readthrough has also been identified in Mimivirus and Megavirus which are the best characterized representatives of an expanding new family of giant viruses infecting Acanthamoeba (64).

Interestingly, in our mutagenesis studies, when the CARYYA motif is mutated by a single nucleotide from CAAUUA to CUAUUA in an otherwise *OPRL1* context thus partially creating the CUAG motif, then wild-type levels of readthrough are restored (Figure 4B). This was not observed in work in a plant system (24) when CAAUUA was similarly mutated to CUAUUA 3′ of a UAG terminator rather than the UGA stop codon used here. Whether this difference reflects a plant versus mammalian difference or different stop codons has not been addressed.

It is interesting that little readthrough was detected in the two candidates with RNA structures (*SACM1L* and *ACP*). Perhaps these structures are binding sites for RNA binding proteins that influence readthrough only in certain cell types, different from the one tested. This could also explain why studies in a heterologous yeast system (33) detected little readthrough for *SACM1L* and C18B2.6, the latter being a worm readthrough candidate with a stem loop.

Our analysis of the effects of aminoglycosides on readthrough sequences with either the CARYYA motif or the CUAG motif indicates that each motif responds differently and suggests that the context effects of patients with premature stop codons could respond differently to individual drugs. A simple test of a specific stop codon and their contexts could be performed before treatment to ascertain the most effective treatment.

## SUPPLEMENTARY DATA

Supplementary Data are available at NAR Online.

SUPPLEMENTARY DATA
