# Supplementary material for: Evidence of efficient stop codon readthrough in four mammalian genes
Source: Nucleic Acids Res. 2014 Jul 10;42(14):8928–38. doi: 10.1093/nar/gku608 (PMC4132726; doi:10.1093/nar/gku608)
Supplement: SUPPLEMENTARY DATA [file supp_42_14_8928__index.html]

Evidence of efficient stop codon readthrough in four mammalian genes — Evidence of efficient stop codon readthrough in four mammalian genes — SUPPLEMENTARY DATA 

# Evidence of efficient stop codon readthrough in four mammalian genes

## SUPPLEMENTARY DATA

**Files in this Data Supplement:**

- Supplementary Data 1
- Supplementary Data 2
