# Supplementary material for: Evidence of efficient stop codon readthrough in four mammalian genes
Source: Nucleic Acids Res. 2014 Jul 10;42(14):8928–38. doi: 10.1093/nar/gku608 (PMC4132726; doi:10.1093/nar/gku608)

Supplementary Figure 1. Comparative evidence for 3 new readthrough candidates: (A-C) Genomic alignments of the readthrough extensions of *ACP2*, *AQP4*, and *MAPK10* with other eutherian mammal genomes, colour coded using CodAlignView ("CodAlignView: a tool for visualizing protein-coding constraint", I Jungreis, M Lin, M Kellis, in preparation). The high concentrations of synonymous substitutions (light green) and conservative amino acid changes (dark green), and relatively low concentrations of radical amino acid changes (red) and frame shifted regions (orange) is characteristic of protein-coding regions. PhyloCSF scores for these extensions are higher than 99% of similar-sized non-coding regions, indicating that they have been functional at the amino acid level in much of the eutherian mammal tree. Horizontal lines in the *ACP2* alignment indicate paired bases in the predicted RNA stem loop. (D) Conserved RNA stem loop 3' of the UGA stop codon in *ACP2*, as predicted by RNAz.

Supplementary Figure 2. Per-codon PhyloCSF scores of the predicted readthrough extensions of the seven readthrough candidates (blue stars) relative to the distributions for coding and non-coding regions. Non-coding distribution was computed from "third ORFs", i.e., the region from the first in-frame stop codon after an annotated stop codon to the next in-frame stop codon, excluding regions that overlap the coding region of another annotated transcript. Coding distribution was computed from same-sized regions directly 5' of the annotated stop codon. Regions less than 10 codons or for which the aligned species encompass less than 10% of the branch length of the 29-mammals tree were excluded because PhyloCSF has insufficient power on such regions. All seven readthrough regions have scores near the middle of the coding distribution and higher than 99.96% of non-coding regions.

Supplementary Figure 3. Nucleotide and codon alignments of the regions corresponding to the end of the main ORF, the readthrough ORF and the flanking sequences of (A) *AQP4*, (B) *MAPK10*, (C) *OPRK1*, (D) *OPRL1* and (E) *SACM1L*. The alignments in A, B, C and D were generated by custom python scripts and clustalW2. First the given region in the main ORF was conceptually translated into a peptide sequence. The conceptual peptide sequences were aligned using ClustalW2. This amino acid alignment was back-translated to a corresponding nucleotide alignment based on codons corresponding to amino acids in the alignment using the initial nucleotide sequences. Finally, the common ancestral (root) sequence was inferred using PyCogenet python module v. 1.5.3 (1) using a ClustalW guide tree. The alignment shown in E used a variant of the procedure for A-D where an alignment was performed directly on the nucleotide sequence with ClustalX2. This nucleotide alignment was then manually adjusted and converted into a corresponding protein sequence alignment which was used to generate a guide tree and evaluate the root sequence as described earlier. Synonymous and non-synonymous substitutions were determined relative to the inferred root sequence shown on top of each alignment. In these alignments, consensus (root) codons (and gaps) are highlighted in white, synonymous codons are highlighted in green, non-synonymous codons are highlighted in brown and stop codons are highlighted in magenta. Abbreviated species identifiers of the sequences used are indicated on the left. Full names of the species whose sequences are used in A-E: *Ailuropoda melanoleuca, Alligator mississippiensis, Anolis carolinensis, Bos Taurus, Callithrix*

jacchus, Callorhinchus milii, Camelus ferus, Canis lupus familiaris, Ceratotherium simum, Chrysemys picta bellii, Condylura cristata, Cricetulus griseus, Danio rerio, Dasypus novemcinctus, Dicentrarchus labrax, Elephantulus edwardii, Equus caballus, Erinaceus europaeus, Felis catus, Ficedula albicollis, Gadus morhua, Gallus gallus, Gasterosteus aculeatus, Geospiza fortis, Haplochromis burtoni, Homo sapiens, Latimeria chalumnae, Lepisosteus oculatus, Leucoraja erinacea, Lipochromis sp., Loxodonta africana, Macaca mulatta, Macropus eugenii, Maylandia zebra, Mchenga conophoros, Meleagris gallopavo, Melopsittacus undulates, Monodelphis domestica, Mus musculus, Mustela putorius furo, Myotis davidii, Neolamprologus brichardi, Nomascus leucogenys, Ochotona princeps, Odobenus rosmarus, Oncorhynchus mykiss, Orcinus orca, Oreochromis niloticus, Ornithorhynchus anatinus, Orycteropus afer, Oryctolagus cuniculus, Oryzias latipes, Otolemur garnettii, Ovis aries, Pelodiscus sinensis, Perca flavescens, Pimephales promelas, Procavia capensis, Pteropus alecto, Pteropus vampyrus, Pundamilia nyererei, Python molurus, Rattus norvegicus, Saimiri boliviensis boliviensi, Salmo salar, Sarcophilus harrisii, Sus scrofa, Taeniopygia guttata, Takifugu rubripes, Tarsius syrichta, Tetraodon nigroviridis, Torpedo californica, Trichechus manatus, Tursiops truncates, Vicugna pacos, Xenopus laevis, Xenopus tropicalis, Xiphophorus maculates. The sequences used in the alignments here were obtained from GenBank using the BLAST (tblastn) algorithm where the translated human sequence was used as bait. The target databases during this search were Expressed Sequence Tags (EST), the Whole-Genome Shotgun contigs, and Transcriptome Shotgun Assembly. All non-mammalian orthologous sequences that could be identified, as well as representative number of mammalian sequences were collected and used in the alignments.

Supplementary Figure 4. Amino acid conservation of OPRK1 and OPRL1 readthrough extensions. The conservation is shown as amino acid logogram (A) of the OPRK1, and (B) the ORPL1 readthrough extensions, respectively. The five highly conserved amino acids near their C-terminal ends, that also show sequence similarity between the two paralogs, are boxed in read. To build the conservation plots the nucleotide sequence alignment for the *OPRK1* and *OPRL1* readthrough extensions (Supplementary Figures 3C and D) were converted into amino acid sequence alignments and used to generate logogram utilizing WebLogo (2).

Supplementary Figure 5. (a) Dual luciferase assay indicating readthrough efficiencies of *OPRL1* constructs with only one 5' nt and with 3' nested deletions showing the importance of the nt immediately 3'. (B) The effect of nt identity immediately 5' of the *OPRL1* UGA on readthrough efficiency. (C) The effect of replacing the UGA stop codon with either UAA or UAG on the readthrough efficiencies of wild type *OPRL1* and *OPRL1*-CARYYA.

REFERENCES

1. Knight, R., Maxwell, P., Birmingham, A., Carnes, J., Caporaso, J.G., Easton, B.C., Eaton, M., Hamady, M., Lindsay, H., Liu, Z. *et al*. (2007) PyCogent: a toolkit for making sense from sequence.Genome Biol., 8, R171.

2. Crooks, G.E., Hon, G., Chandonia, J.M. and Brenner, S.E. (2004) WebLogo: a sequence logo generator. Genome Res., 14, 1188-1190.

Supplementary Figure 1A

Human_aa	D	H	A	*	Q	P	L	S	P	L	P	S	T	S	*	G	R	W	A	G	P	S
Paired RNA							—	—	—	—	—	–			—	—	—	—	—	—		
ancestor	GAC	CAT	GCC	TGA	CAA	CCA	CTC	AGC	CCC	CTT	CCC	TCT	GCC	TCC	TAG	GGG	AGG	TGG	GCT	GGG	CCC	TTG
Human	GAC	CAC	GCC	TGA	CAA	CCA	CTC	AGC	CCC	CTT	CCC	TCC	ACC	TCC	TAG	GGG	AGG	TGG	GCT	GGG	CCC	TCG
Chimp	GAC	CAC	GCC	TGA	CAA	CCA	CTC	AGC	CCC	CTT	CCC	TCC	ACC	TCC	TAG	GGG	AGG	TGG	GCT	GGG	CCC	TCG
Rhesus	GAC	CAT	GCC	TGA	CAA	CCA	CTC	AGC	CCG	CTT	CCC	TCC	GCC	TCC	TAG	GGG	AGG	TGG	GCT	GGG	CCC	TCG
Tarsier	GAC	CAT	GCC	TGA	CAA	CCA	CTC	AGC	CCT	CTT	CCC	TCT	GCC	TCC	TAG	GGG	AAG	TGG	GCT	GG-	CCC	TTG
Mouse_lemur	GAC	-AT	GC-	TGA	CAG	C-A	CTC	AGC	CCG	CTT	CCC	TCT	GC-	TCC	TAG	GGG	AGG	TGG	GCT	GGG	CCC	TTG
Bushbaby	GAC	CAT	GCC	TGA	CAA	CCA	CTC	AGC	CCG	CTT	CCC	TCT	ACC	TCC	TAG	GGG	AGG	TGG	GCT	GGG	CCT	TTG
TreeShrew	GAC	CAT	GCC	TGA	CAC	CCA	GCT	GGC	CCC	CTT	CCC	TCT	GCC	TCC	TAG	GGG	AGG	AGG	GCT	GGG	CCC	TTG
Mouse	GAC	CAT	GCT	TGA	CAA	CCA	CTC	AGT	CCC	CTT	CCC	TCT	GCC	TCC	TAG	GGG	AGG	TGG	GCT	GAG	TCC	TCG
Rat	GAC	CAT	GCT	TGA	CAA	CCA	CTC	AGT	CCC	CTT	CCC	TCT	GCC	TCC	TAG	GGG	ATG	TGG	GCT	GAG	TCC	TTG
Kangaroo_rat	GAT	CAT	GCC	TGA	CAA	CCA	CTC	AGC	CCC	CTT	CCC	TCT	GCC	TCC	TAG	GGG	AGG	TGG	GCT	GGG	CCC	TTC
Guinea_Pig	GAC	CAC	GCC	TGA	CAA	CCA	CTC	AGC	CCC	CTT	CCC	GCT	GCC	TCC	TAG	GGA	A-G	TGG	GCT	GG-	-CC	CCG
Squirrel	GAC	CAT	GCC	TGA	CAA	CCA	CTC	AGC	CCC	CTT	CCC	TCT	GCC	TCC	TAG	GGG	AGG	TGG	GCT	GGG	-CC	CTG
Rabbit	GAC	CAT	GCC	TGA	CAA	CCA	CTC	AGC	CCC	CTT	CCC	TCC	GCC	TCC	TAG	GGG	AGG	TGG	GCT	GG-	CCC	TTG
Pika	GAC	CGC	GCC	TGA	CAA	CCA	CTC	AGC	CCC	CTC	CCC	TCT	GCC	TGC	TAG	GGG	AGG	TGG	GCT	GGA	CCT	TTG
Dolphin	GAC	CAT	GCC	TGA	CAA	CCA	CTC	AGC	CCC	CTT	CCC	TCT	GCC	TCC	TCG	GGG	AGG	TGG	GCT	GGG	CC-	TTG
Cow	GAC	CAC	GCC	TGA	CAA	CCA	CTC	AGC	CCC	CTT	CCC	TCT	GCC	TCC	TAG	GGG	AGG	TGG	GCT	GGG	CCC	TTG
Horse	GAT	CAC	GCC	TGA	CAA	CCA	CTC	AGC	CCC	CTT	CCC	TCT	GCC	TCC	TAG	GGG	AGG	TGG	GCT	GGG	CCC	TTG
Cat	GAT	CAT	GCC	TGA	CAA	CCA	CTC	AGC	CCC	CTT	CCC	TCT	GCC	TCC	TAG	GGG	AGG	TGG	GCT	GGG	CCC	TTG
Dog	GAT	CAT	GCC	TGA	CAA	CCA	CTC	AGC	CCC	CTT	CCC	TCT	GCC	TCC	TAG	GGG	AGG	TGG	GCT	GGG	CCC	TTG
Microbat	GAT	CAC	GCC	TGA	CAA	CCA	CTC	AGC	CCC	CTT	CCC	TCT	GCC	TCT	TAG	GGG	AGG	TGG	GCT	TGG	CCC	TCA
Megabat	GAT	CAC	GCC	TGA	CAA	CCA	CTC	AGC	CCC	CTT	CCC	TCT	GCC	TCC	TAG	GGG	AGG	TGG	GCT	TGG	CCC	TTA
Hedgehog	GAC	CAC	GCC	TGA	CAA	CCA	CTC	AGC	CCC	CTT	CCC	TCT	GCC	TCC	TAG	GGG	AGG	TGG	GCT	GGG	CCC	CTG
Shrew	GAC	CAC	GCC	TGA	CAA	CCG	CTC	AGC	CCT	CTT	CCC	TCG	GCC	TCC	TAG	GGG	AGG	TGG	GCT	G--	---	---
Elephant	GAC	CAT	GCC	TGA	CAG	CTA	CTC	AGC	CCC	CTT	CCC	TCT	GCC	TCC	TAG	GGG	AGG	TGG	GCT	GGG	CCC	TTG
Rock_hyrax	GAC	CAC	GCC	TGA	CAA	CCA	CTC	AGC	CCC	CTT	CCC	TCT	GCC	TCC	TAG	GGG	AGG	TGG	GCT	GGG	CCC	CTG
Tenrec	GAC	CAT	GCC	TGA	CAA	CCA	CT-	GGC	CCC	CTT	CCC	TCT	GCC	TCC	TAG	GGG	AGG	CGG	GCT	GGG	CCC	TTG
Armadillo	GAC	CAT	GCC	TGA	CAC	CTA	CTC	CGC	CCC	CTT	CCT	TCT	GTC	TCC	TAG	GGG	AG-	---	---	---	---	---

ACP2

No Change	GAC
Synonymous	GAT
Conservative	GAA
Radical	GGG
TAA Stop	TAA
TAG Stop	TAG
TGA Stop	TGA
Indel	GA-
Frameshifted	GAC

Supplementary Figure 1B

Human_aa	S	S	V	*	L	E	D	R	T	E	S	R	Q	D	S	L	E	L	S
ancestor	TCT	TCA	GTA	TGA	CTA	GAA	GAT	AGC	ACT	GAA	AGC	AGA	CGA	GAA	CCC	TTA	GAA	CTG	TCC
Human	TCT	TCA	GTA	TGA	CTA	GAA	GAT	CGC	ACT	GAA	AGC	AGA	CAA	GAC	TCC	TTA	GAA	CTG	TCC
Chimp	TCT	TCA	GTA	TGA	CTA	GAA	GAT	CGC	ACT	GAA	AGC	AGA	CAA	GAC	TCC	TTA	GAA	CTG	TCC
Rhesus	TCT	TCA	GTA	TGA	CTA	GAA	GAT	CGC	ACT	GAA	AGC	AGA	CAA	GAC	TCC	TTA	GAA	CTG	TCC
Tarsier	TCT	TCA	GTA	TGA	CTA	GAA	GAC	AGC	ACT	GAA	AAC	AGA	CGA	GAC	ACC	TTA	GAA	TTG	TCC
Mouse_lemur	TGT	CTC	GTA	TGA	CTA	GGA	GAC	AGC	ACT	GA-	AGC	AGA	---	---	---	---	---	---	...
Bushbaby	TCT	TCA	GTA	TGA	CTA	GAA	GAC	AGC	GCT	GAG	AGC	AGA	CGA	GAC	TCC	CTA	GAA	CTG	TCC
TreeShrew	TCT	TCA	GTA	TGA	CTA	GAA	GAT	AGC	ACT	GAG	AGC	AGA	CGA	GAC	CCC	TTA	GAA	CTG	TCC
Mouse	TCT	TCC	GTA	TGA	CTA	GAG	GAC	AGC	ACT	GAA	GGC	AGA	AGA	GAC	TCC	CTA	GAC	CTG	GCC
Rat	TCT	TCT	GTA	TGA	CTA	GAG	GAC	AGC	ACT	GAA	AAC	AGA	AGA	GAC	TCC	CTA	GAA	CTG	GCC
Kangaroo_rat	TCT	TCA	GTA	TGA	CTA	GAA	GAT	AGC	ACT	GAA	AGC	AGA	CGA	GAC	CCC	TTA	GAA	CGG	TCC
Guinea_Pig	TCT	TCG	GTA	TGA	CTA	GAA	GAC	GGC	ACT	GAA	AGC	AGA	CGA	GAC	TCC	TTA	GAA	CTG	TCC
Squirrel	TCT	TCA	GTA	TGA	CTA	GAA	GAT	AGC	ACT	GAA	AGC	AGA	AGA	GAA	CCC	TTA	GAA	CGG	TCC
Rabbit	TCT	TCC	GTA	TGA	CTA	GAA	GAA	AGC	ACT	GAA	AGC	AGA	CAA	GAC	CCT	GTG	GAA	GAG	TCC
Pika	TCC	TCA	GTA	TGA	CTA	GAA	GAT	AGC	ACT	GAA	AGC	AGA	CAA	CGT	CC-	--A	GGA	GAG	TCC
Alpaca	TCT	TCA	GTA	TGA	CTA	GAG	GAC	AGC	ACT	GAA	AGC	AGA	CAA	GAA	CCC	TTA	GAA	CTG	TCC
Dolphin	TCT	TCA	GTA	TGA	CTA	GAA	GAT	AGC	ACT	GAA	AGC	AGA	CAA	GAG	CCC	TTA	GAA	CTG	TCC
Cow	TCT	TCA	GTA	TGA	CTA	GCA	GGC	AGC	ACT	GAA	AGC	AGA	CAA	GAG	CCC	TTA	GAA	CTG	TCC
Horse	TCT	TCA	GTA	TGA	CTA	GAA	GAC	AGC	ACT	GAA	AGC	AGA	CAA	GAA	CCC	TTA	GAA	CTG	TCC
Dog	TCA	TCG	GTA	TGA	CTA	GAA	GGT	GGC	ACT	GAA	AAC	AGA	CAG	GAG	CCC	TCA	GAA	CTG	TCC
Microbat	TCC	TCC	GTA	TGA	CTA	GAA	GAT	GGC	ACT	GAA	AGC	AGA	CGC	GAG	CCC	TTA	GAG	CTG	CCC
Megabat	TCT	TCG	GTA	TGA	CTA	GAA	GAT	AGC	ACT	GAA	AGC	AGA	CAA	GAA	CCC	TTA	GAA	CTA	TCC
Shrew	TCT	TCA	GTA	TGA	CTA	GAA	GAC	AGC	ACT	GAA	AAC	AGA	CAA	GAG	CCC	TTC	CAG	CCA	TCC
Elephant	TCT	TCA	GTA	TGA	CTA	GAA	GGT	AGC	ACT	GAA	AGC	AGA	CGG	GAA	CCC	TTA	GAA	CTA	TCC
Rock_hyrax	TCT	TCG	GTA	TGA	CTA	GAG	GAA	AGC	ACT	GAA	AGC	AGA	CGA	GAA	CCC	TTA	GAA	CTA	TCC
Tenrec	TCT	TCC	GTA	TGA	CTA	GAA	GAC	AGC	GCT	GAG	AGC	AGA	CGG	GAA	CCC	GTA	GAC	CTG	GCC
Armadillo	TCT	TCA	GTA	TGA	CTA	GAA	GAT	AGC	ACT	GAA	AGC	AGA	CGA	GGA	CCC	TTA	GAA	CTG	TCC
Sloth	TCT	TCA	GTA	TGA	CTA	GAA	GAT	AGC	ACT	GAA	AGC	AGA	CGA	GAA	CCC	TTA	GAA	CGA	TCC

Human_aa	S	D	F	L	P	P	I	K	E	T	D	L	L	*	I	R	N	V	Q
ancestor	TCA	GAT	TTC	CTT	CCA	CCC	ATT	AAG	GAA	ACA	GAT	TTG	TTA	TAA	ATT	AGA	CAT	GTG	CAG
Human	TCA	GAT	TTC	CTT	CCA	CCC	ATT	AAG	GAA	ACA	GAT	TTG	TTA	TAA	ATT	AGA	AAT	GTG	CAG
Chimp	TCA	GAT	TTC	CTT	CCA	CCC	ATT	AAG	GAA	ACA	GAT	TTG	TTA	TAA	ATT	AGA	AAT	GTG	CTG
Rhesus	TCA	GAT	TTC	CTT	CCA	CCC	ATT	AAG	GAA	ACA	GAT	TTA	TTA	TAA	ATT	AGA	CAT	GTG	CAG
Tarsier	TCA	GAT	TTT	CTT	CCA	CCC	ATT	AAG	GAA	ACA	GAT	TTG	TTA	TAA	ATT	AGA	CAT	GTG	CAG
Mouse_lemur	...	...	...	...	...	...	...	...	...	...	...	...	...	...	...	...	...	...	...
Bushbaby	TCA	GAT	TTC	CTT	CCA	CCC	ATT	AAG	GAA	ACA	GAT	TTG	TTA	TAA	ATT	AGA	CAC	GAG	CAG
TreeShrew	TCA	GAT	TTC	CTC	CCA	CCC	ATT	AAG	GAA	ACA	GAT	TTG	TTA	TGA	ATT	AGA	CAC	GTA	CAG
Mouse	TCA	GAT	TTC	CTG	CCA	CCC	ATT	AAG	GAA	ACA	GAT	TTG	TTA	TAA	ATT	AGA	CAC	TTG	CGG
Rat	TCA	GAT	TTC	CTG	CCA	CCC	ATT	AAG	GAA	ACA	GAT	TTG	TTA	TAA	ATT	AGA	CAT	ATG	CGG
Kangaroo_rat	TCA	GAT	TTC	CTT	CCC	CCT	ATC	AAG	GAG	ACA	GAT	TTG	TTG	TAA	ATT	AGA	CAC	ATG	CAG
Guinea_Pig	TCA	GAT	TTC	CTT	CCG	CCC	ATT	AAG	GAA	ACA	GAT	TTG	TTG	TAA	GTT	AGA	CAC	AAG	CAG
Squirrel	TCA	GAT	TTC	CTT	CCA	CCC	ATT	AAG	GAG	ACA	GAT	TTG	TTA	TAA	ATT	AGA	CAT	GTG	CAG
Rabbit	TCA	GAT	TTC	CTT	CCA	CCC	ATT	AAG	GAA	ACA	GAT	TTG	TTA	TAA	ATT	GGG	CAT	GTA	CAG
Pika	TCA	GAT	TTC	CTT	CCA	CCC	ATT	AAG	GAA	ACA	GAT	TTG	TTA	TGA	ATT	AGG	CAT	GTG	CAG
Alpaca	TCA	GAT	TTC	CTT	CCA	CCC	ATT	AAG	GAA	ACA	GAT	CTG	TTA	TAA	ACT	AGA	CAT	GTA	CAG
Dolphin	TCA	GAT	TTT	CTT	CCG	CCC	ATT	AAG	GAA	ACA	GAT	TTG	TTA	TAA	ATT	AGA	CAT	GTG	CAG
Cow	TCA	GAT	CTC	CTT	CCA	CCC	ATT	AAG	GAA	ACA	GAT	TTG	TTA	TAA	ATT	AGA	CAT	GTG	CAG
Horse	TCA	GAT	TTC	CTC	CCA	CCC	ATT	AAG	GAA	ACA	GAT	TTG	TTA	TAA	ATT	AGA	CAC	GTG	CAG
Dog	TCA	GAT	TTC	CTT	CCA	CCC	ATT	AAG	GAA	ACA	GAT	TTG	TTA	TAA	ATT	AGA	CAC	GCG	CAG
Microbat	GCA	GAG	GTC	CTC	CCG	CCC	ATT	AAG	GAA	ACA	GAC	TTG	TTA	TAA	GGT	AGA	CGT	GT-	---
Megabat	TCA	GAT	TTC	CTT	CCA	CAC	ATT	AAG	GAA	ACA	GAT	TTG	TTA	TAA	ATT	AGA	CAT	GTG	CAG
Shrew	TCA	GAT	TTC	CTT	CCA	CCC	ATT	AAG	GAA	ACA	GAT	TTG	CTA	TAA	ATG	AGA	CAT	GTG	CAG
Elephant	TCA	GAT	TTC	CTT	CCA	CCC	ATT	AAA	GAA	ACA	GAT	TTG	TTA	TAA	-CT	AGC	CCT	GT-	-AA
Rock_hyrax	TCA	GAT	TTC	CTT	CCA	CCC	ATT	AAA	GAA	ACA	GAT	TTG	TTG	TAA	-CT	AGA	TAT	GTG	CAG
Tenrec	TCA	GAT	TTC	CTT	CCA	CCC	ATT	AAA	GAA	ACA	GAT	TTG	CTA	TAA	-TT	AGA	CAC	GTG	CAG
Armadillo	TCA	GAT	TTC	CTT	CCA	CCC	ATT	AAG	GAA	ACA	GAT	TTG	TTA	TAA	GTT	AGA	CAT	GAG	CAG
Sloth	ACA	GAT	TAC	CTT	CCA	CCC	ATT	AAG	GAA	ACA	GAT	TTG	TTA	TGA	ATT	AGA	CAT	GAG	CAG

AQP4

No Change	GAC
Synonymous	GAT
Conservative	GAA
Radical	GGG
TAA Stop	TAA
TAG Stop	TAG
TGA Stop	TGA
Indel	GA-
Frameshifted	GAC

Supplementary Figure 1C

```
  Human_aa C   C   R   *   L   A   A   C   L   R   N   P   A   F   F   R   R   *     C       D    G      T
  ancestor TGT TGC AGG TGA CTA GCC GCC TGC CTG CGA AAC CCA GCG TTC TTC AGG AGA TGA TG---T--- GAT GGA---- ACA
     Human TGT TGC AGG TGA CTA GCC GCC TGC CTG CGA AAC CCA GCG TTC TTC AGG AGA TGA TG---T--- GAT GGA---- ACA
     Chimp TGT TGC AGG TGA CTA GCC GCC TGC CTG CGA AAC CCA GCG TTC TTC AGG AGA TGA TG---T--- GAT GGA---- ACA
    Rhesus TGT TGC AGG TGA CTA GCC GCC TGC CTG CGA AAC CCA GCG TTC TTC AGG AGA TGA TG---T--- GAT GGA---- ACA
   Tarsier TGT TGC AGG TGA CTA GCC GCC TGC CTG CGA AAT CCA GCG TTC TTC AGG AGA TGA TG---T--- GAT GGA---- ACA
Mouse_lemur TGT TGC AGG TGA CTA GCC GCC TGC CTG CGA AAC CCA GCG TTC TTC AGG AGA TGA CG---T--- GAT GGA---- GCA
  Bushbaby TGT TGC AGG TGA CTA GCC GCC TGC CTG CGA AAC CCA GCG TTC TTC AGG AGA TGA TG---T--- GAT GGA---- ACA
 TreeShrew TGT TGC AGG TGA CTA GCC GCC TGC CTG CGA AAC CCA GCG TTC TTC AGG AGA TGA TG---A--- GAT GGA---- ACA
     Mouse TGT TGC AGG TGA CTA GCC GCC TGC CTG CGA AAC CCA GCG TTC TTC AGG AGA TGA CG---C--- GAT AGA---- ACA
       Rat TGT TGC AGG TGA CTA GCC GCC TGC CTG CGA AAC CCA GCG TTC TTC AGG AGA TGA CG---CCAT GAT AGA---- ACA
Kangaroo_rat TGC TGC AGG TGA CTA GCC GCC TGC CTG CGA AAC CCA GCA TTC TTC AGG AGA TGA TA---C--- GAT AGA---- ACA
Guinea_Pig TGT TGC AGG TGA CTA GCC GCC TGC TTG CGG AAC CCA GCG TTC TTC AGG AGC TGA GG---G--- GAT GGA---- ACA
  Squirrel TGT TGC AGG TGA CTA GCC GCC TGC CTG CGA AAC CCA GCG TTC TTC AGG AGA TGA TG---T--- GAT GGA---- ACA
    Rabbit TGT TGC AGG TGA CTA GCC GCC TGC CTG CGA AAC CCA GCG TTC TTC AGG AGA TGA TG---T--- GAT GGA---- ACA
      Pika TGT TGC AGG TGA CTA GCC GCC TGC CTG CGA AAC CCA GCG TTC TTC AGG AGA TGA TGATGT--- GAT GGA---- ACA
    Alpaca TGT TGC AGG TGA CTA GCC GCC TGC CTG CGA AAC CCG GCG TTC TTC AGG AGA TGA TG---T--- GAT GGA---- ACA
   Dolphin TGT TGC AGG TGA CTA GCC GCC TGC CTG CGA AAC CCA ACG TTC TTC AGG AGA TGA TG---T--- GAT GGA---- ACA
       Cow TGT TGC AGG TGA CTA GCC GCC TGC CTG CGA AAC CCA GCG TTC TTC AGG AGA TGA TG---T--- GAT GGA---- ACA
     Horse TGT TGC AGG TGA CTA GCC GCC TGC CTG CGA AAC CCA GCG TTC TTC AGG AGA TGA TG---T--- GAT GGAGCAC ACA
       Cat TGT TGC AGG TGA CTA GCC GCC TGC CTG CGA AAC CCA GCA TTC TTC AGG AGA TGA TG---C--- GAT GGA---- ACA
       Dog TGT TGC AGG TGA CTA GCC GCC TGC CTG CGA AAC CCA GCG TTC TTC AGG AGA TGA TG---T--- GAT GGA---- ACA
  Microbat TGT TGC AGG TGA CTA GCC GCC TGC CTG CGA AAC CCA GCG TTC TTC AGC AGA TGA TG---G--- GAT GGA---- ACA
   Megabat TGT TGC AGG TGA CTA GCC GCC TGC CTG CGA AAC CCA GCG TTC TTC AGG AGA TGA TG---C--- GAT GGG---- ACA
  Hedgehog TGT TGC AGG TGA CTA GCC GCC TGC CTG AGA AAC CCA GCG TTC TTC AGG AGA TGA CA---T--- GAC GGA---- ACA
     Shrew TGT TGC AGG TGA CTA GCC GCC TGC CTG CGA AAC CCA GCG TTC TTC AGG AGA TGA CA---T--- GAT GGA---- ACA
  Elephant TGT TGC AGG TGA CTA GCC GCC TGC CTG CGA AAC CCA GCG TTC TTC AGG AGA TGA TG---C--- GAT GGA---- ACA
Rock_hyrax TGT TGC AGG TGA CTA GCC GCC TGC CTG CGA AAC CCA GCG TTC TTC AGG AGA TGA TG---C--- GAT GGA---- ACA
    Tenrec TGT TGC AGG TGA CTA GCC GCC TGC CTG CGA AAC CCA GCG TTC TTC AGG AGA TGA TG---C--- GAT GGG---- ATA
 Armadillo TGT TGC AGG TGA CTA GCC GCC TGC CTG CGA AAC CCA GCG TTC TTC AGG AGA TGA TG---G--- GAT GGA---- ACA
     Sloth TGT TGC AGG TGA CTA GCC GCC TGC CTG CGA AAC CCA GCG TTC TTC AGG AGA TGA TG---T--- GAT GGA---- ACA
```

MAPK10

No Change	GAC
Synonymous	GAT
Conservative	GAA
Radical	GGG
TAA Stop	TAA
TAG Stop	TAG
TGA Stop	TGA
Indel	GA-
Frameshifted	GAC

Supplementary Figure 1D

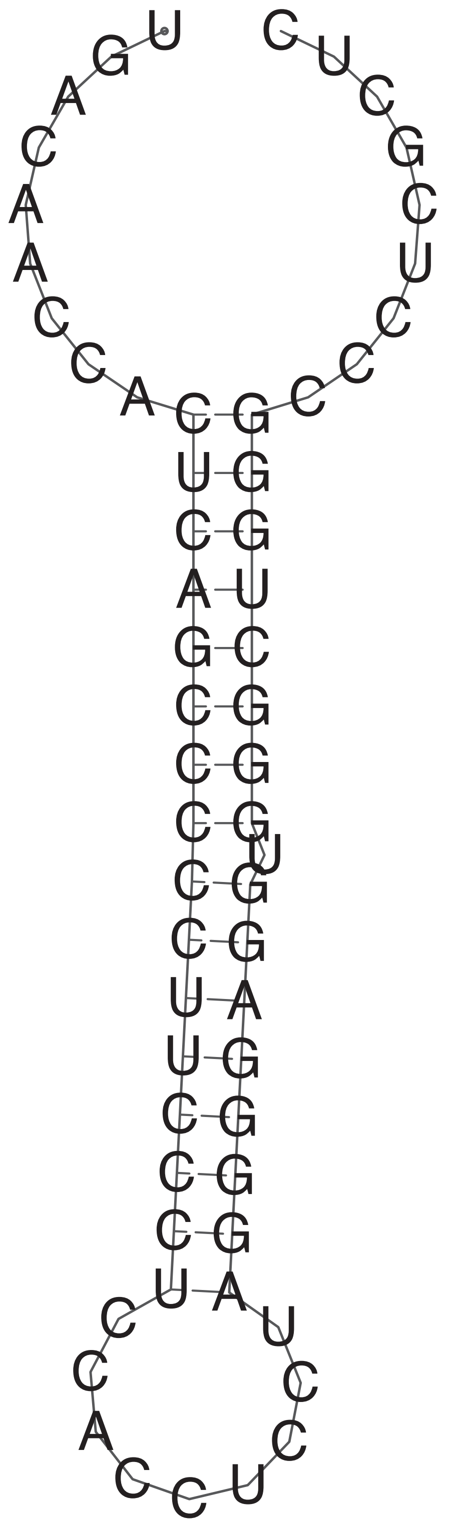

Supplementary Figure 2

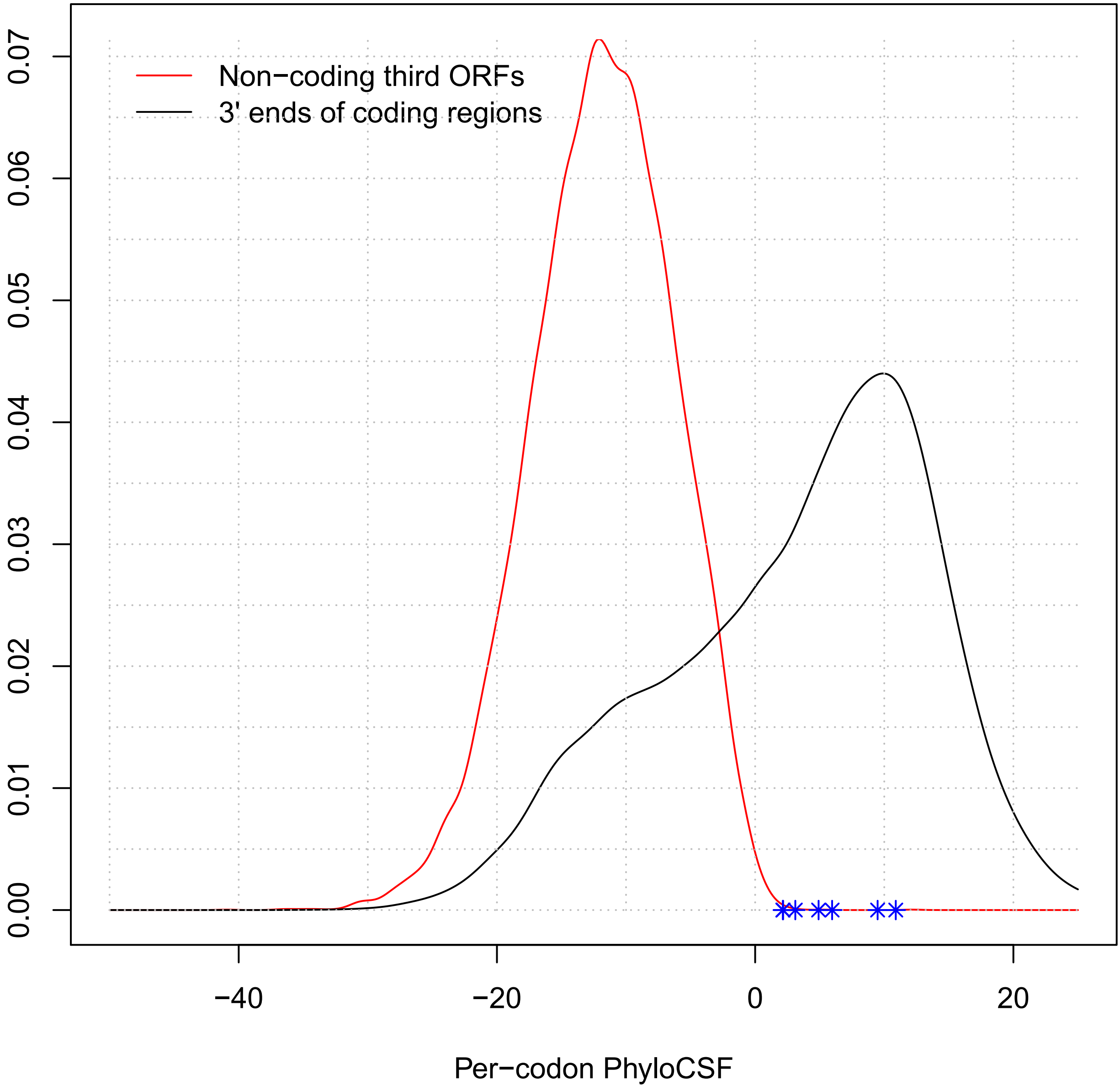

Supplement: SUPPLEMENTARY DATA [file supp_gku608_nar-00627-a-2014-File008.pdf]
