# Supplementary material for: Evidence of efficient stop codon readthrough in four mammalian genes
Source: Nucleic Acids Res. 2014 Jul 10;42(14):8928–38. doi: 10.1093/nar/gku608 (PMC4132726; doi:10.1093/nar/gku608)

Supplementary Figure 3A

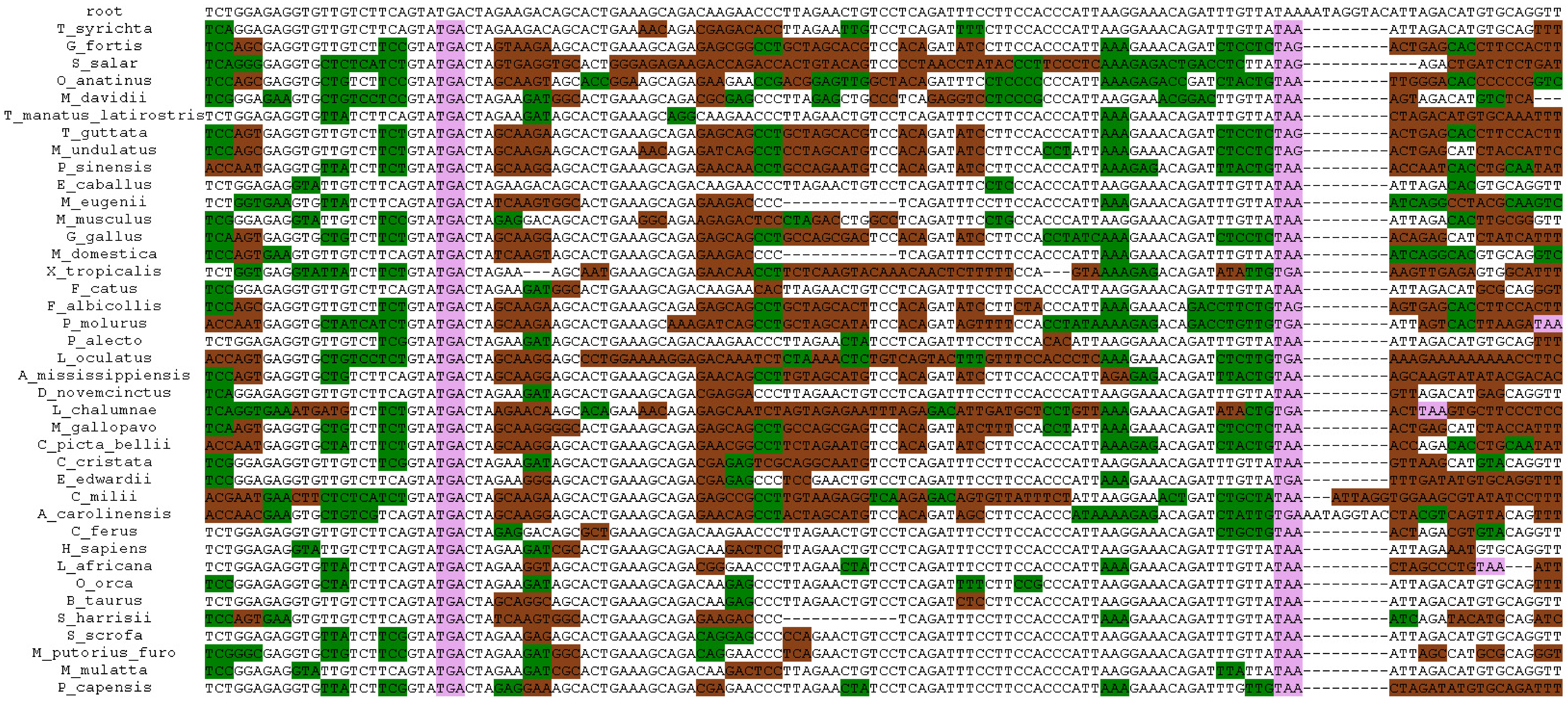

Supplementary Figure 3B

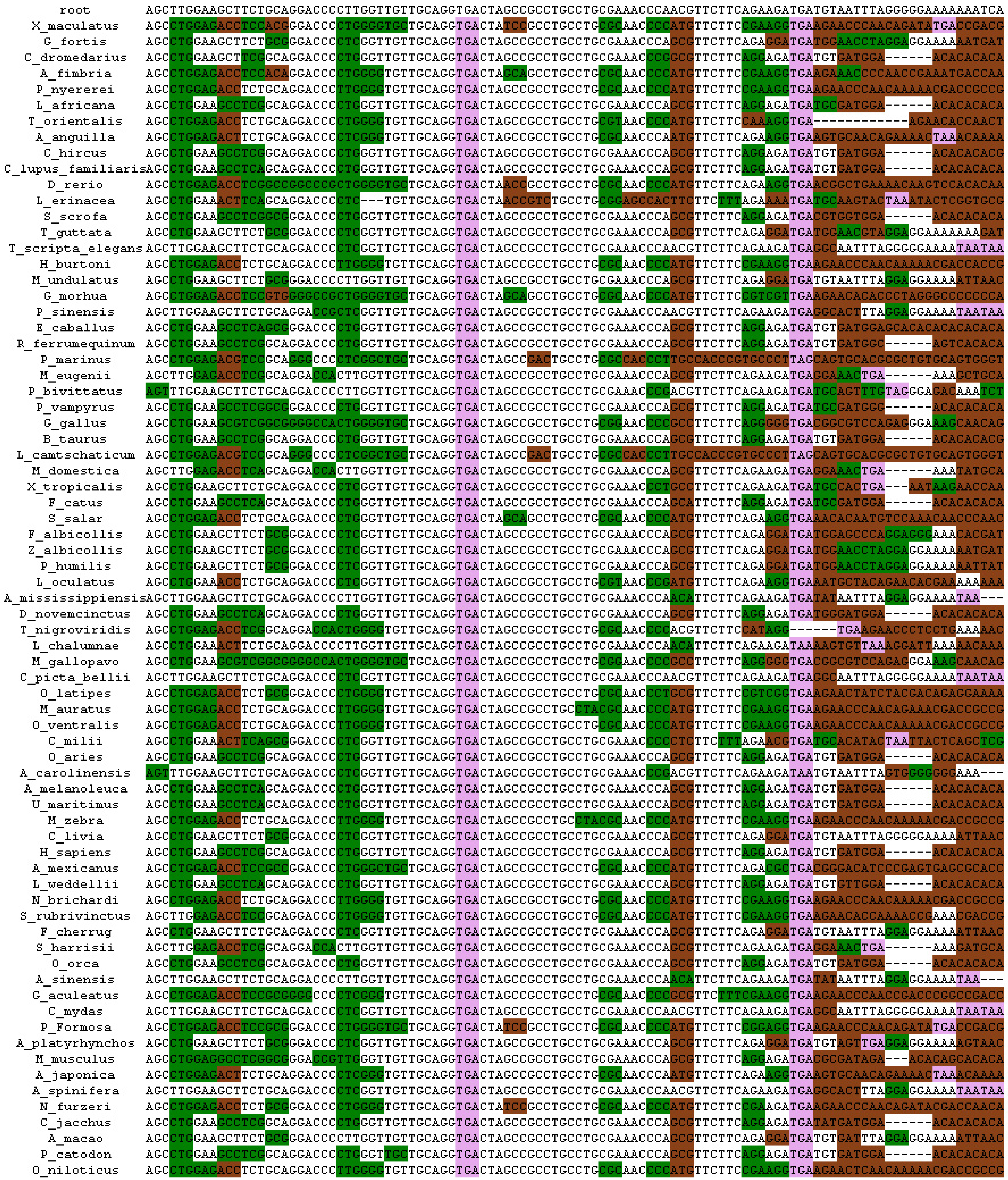

Supplementary Figure 3C

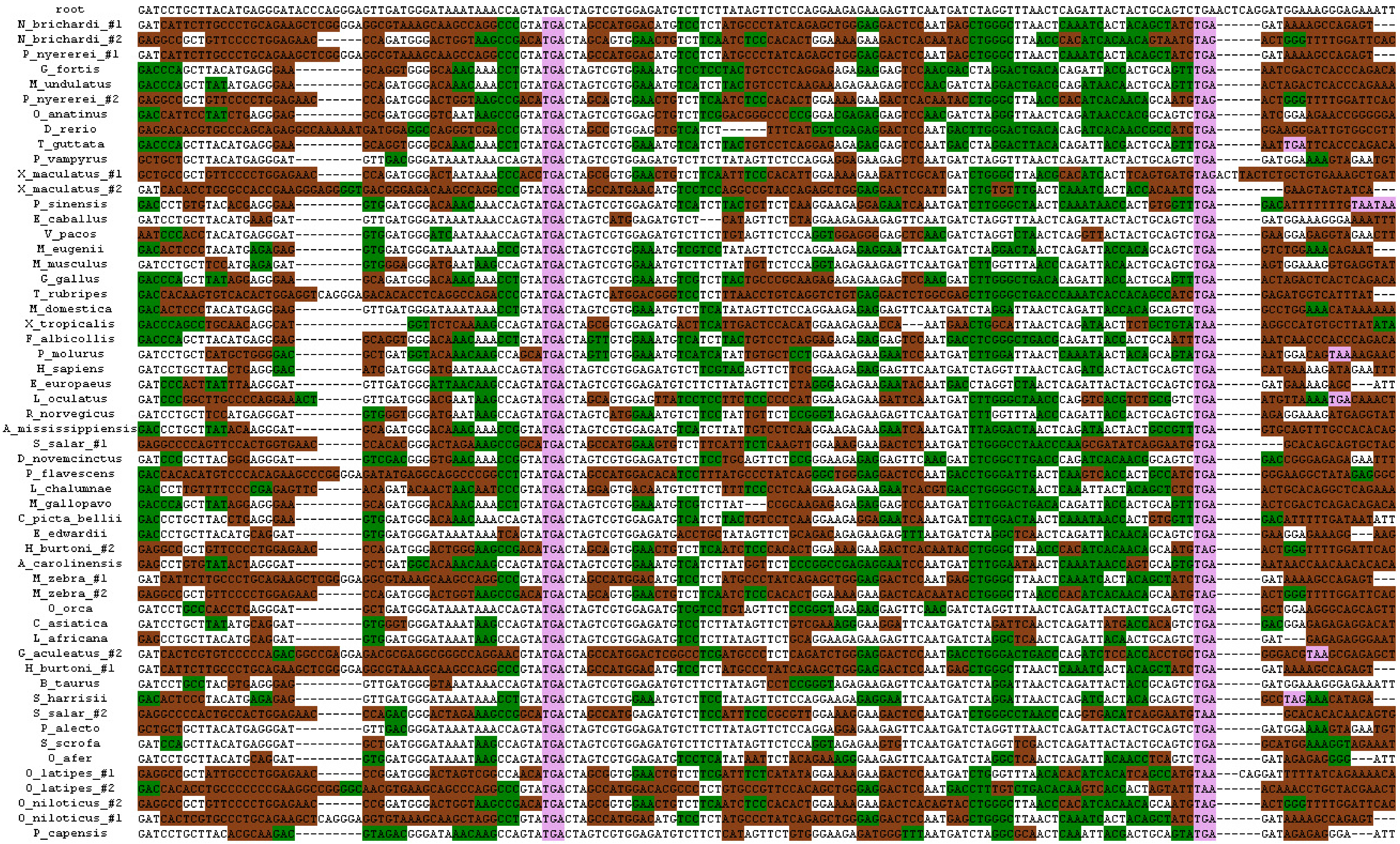

Supplementary Figure 3D

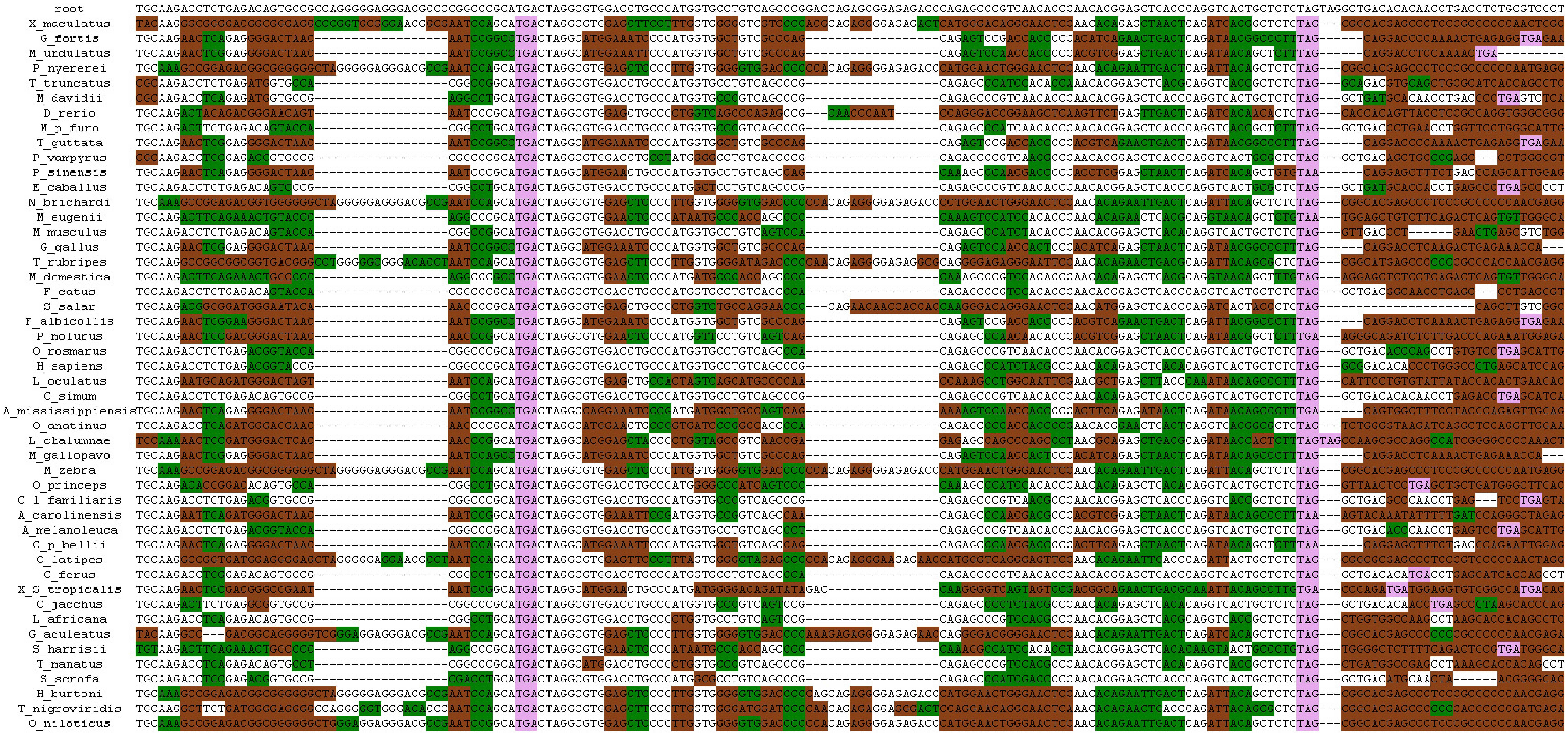

Supplementary Figure 3E

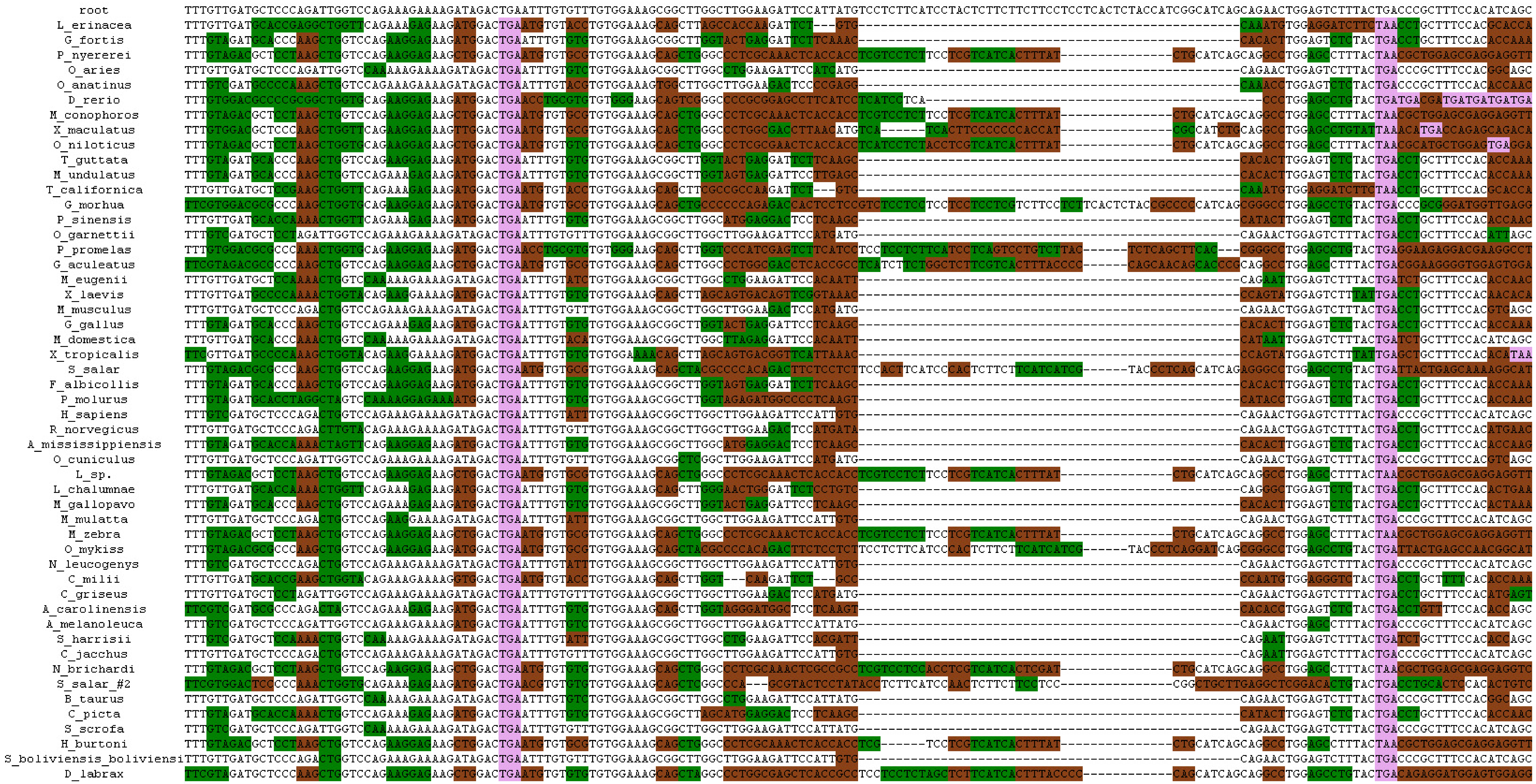

Supplementary Figure 4

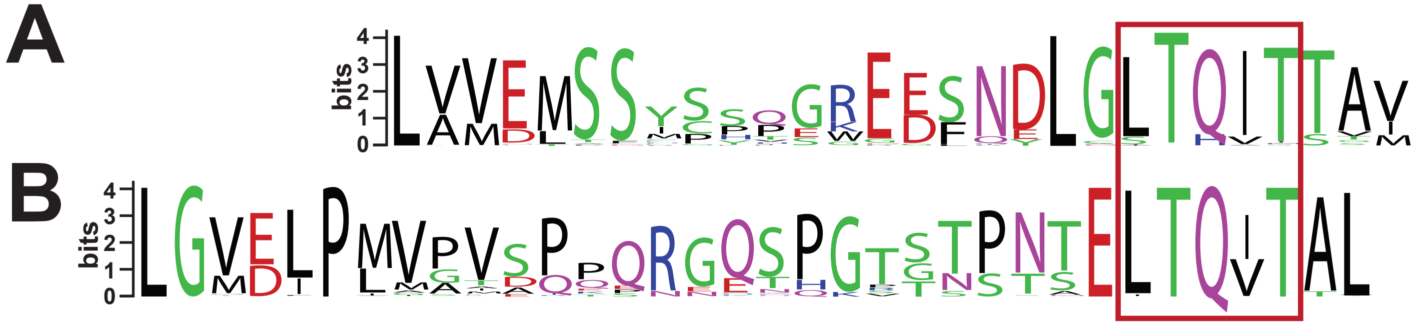

Supplementary Figure 5

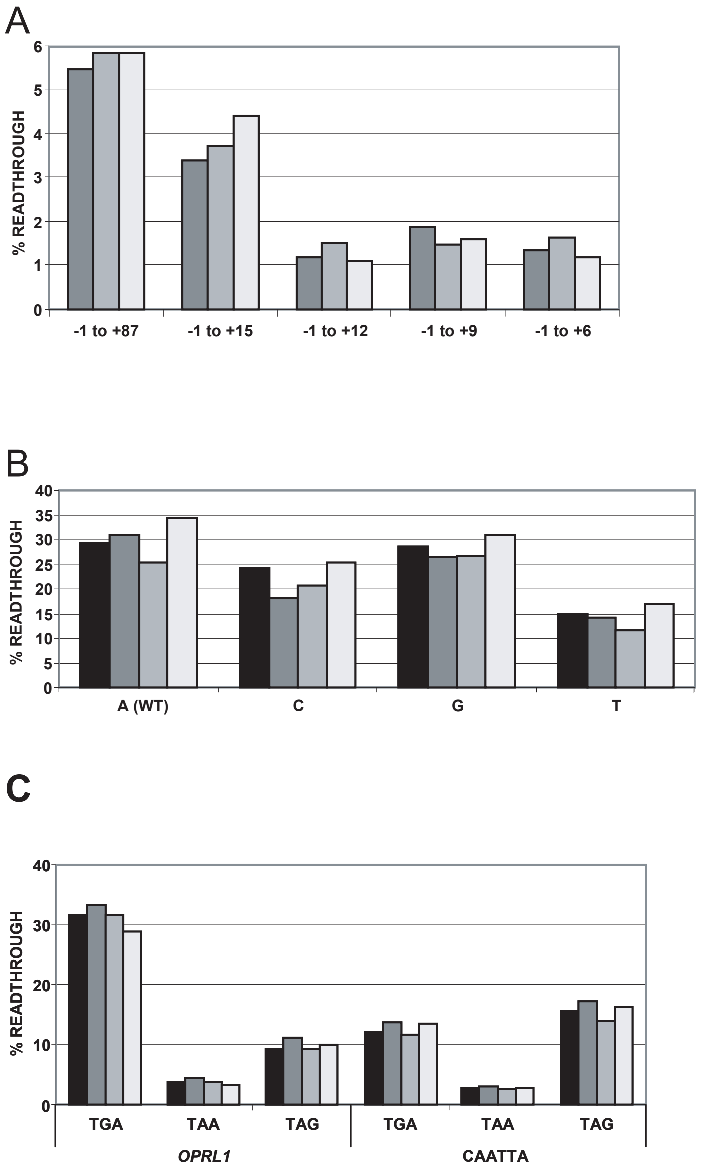

Supplement: SUPPLEMENTARY DATA [file supp_gku608_nar-00627-a-2014-File009.pdf]
